# A Biomimetic, Silaffin R5-Based Antigen Delivery Platform

**DOI:** 10.3390/pharmaceutics15010121

**Published:** 2022-12-29

**Authors:** Daniela Reichinger, Manuel Reithofer, Mariam Hohagen, Mirjana Drinic, Joshua Tobias, Ursula Wiedermann, Freddy Kleitz, Beatrice Jahn-Schmid, Christian F. W. Becker

**Affiliations:** 1 Institute of Biological Chemistry, Faculty of Chemistry, University of Vienna, Währinger Str. 38, 1090 Vienna, Austria; 2 Vienna Doctoral School in Chemistry (DoSChem), University of Vienna, Währinger Str. 42, 1090 Vienna, Austria; 3 Department of Pathophysiology and Allergy Research, Center for Pathophysiology, Infectiology and Immunology, Medical University of Vienna, Währinger Gürtel 18-20, 1090 Vienna, Austria; 4 Institute of Molecular Biotechnology, Department of Biotechnology, University of Natural Resources and Life Sciences, Gregor-Mendel-Straße 33, 1180 Vienna, Austria; 5 Department of Inorganic Chemistry–Functional Materials, Faculty of Chemistry, University of Vienna, Währinger Str. 42, 1090 Vienna, Austria; 6 Institute of Specific Prophylaxis and Tropical Medicine, Center for Pathophysiology, Infectiology and Immunology, Medical University of Vienna, Kinderspitalgasse 15, 1090 Vienna, Austria

**Keywords:** adjuvant, biomineralization, silica particles, biomaterial

## Abstract

Nature offers a wide range of evolutionary optimized materials that combine unique properties with intrinsic biocompatibility and that can be exploited as biomimetic materials. The R5 and RRIL peptides employed here are derived from silaffin proteins that play a crucial role in the biomineralization of marine diatom silica shells and are also able to form silica materials in vitro. Here, we demonstrate the application of biomimetic silica particles as a vaccine delivery and adjuvant platform by linking the precipitating peptides R5 and the RRIL motif to a variety of peptide antigens. The resulting antigen-loaded silica particles combine the advantages of biomaterial-based vaccines with the proven intracellular uptake of silica particles. These particles induce NETosis in human neutrophils as well as IL-6 and TNF-α secretion in murine bone marrow-derived dendritic cells.

## 1. Introduction

Vaccination is among the most important achievements in the history of medicine and has saved countless lives by eradicating [1] or greatly reducing the incidence as well as the severity of diseases such as diphtheria, measles, mumps, and polio infections [2]. The potential devastating effects of diseases for which no vaccine exists yet have been distressingly revealed in the early phase of the recent SARS-CoV-2 crisis. Many of the most effective vaccines today are based on live or attenuated pathogens and sequence information (e.g., mRNA). However, in some cases, this approach is neither practical (e.g., viruses that do not grow well in culture such as hepatitis C virus) nor entirely safe (e.g., viruses that cause chronic infections such as human immunodeficiency viruses) [3,4]. To avoid such risks, subunit vaccines, consisting of recombinant or synthetic antigens, e.g., peptides, pose a promising alternative. These subunit vaccines are more specific, safer and often less expensive to produce compared to live vaccines [3]. However, subunit vaccines typically elicit much weaker and shorter-lasting immune responses and therefore require the addition of an adjuvant and/or delivery vehicle to enhance their immunogenicity and vaccine potency [1,5,6]. Despite testing hundreds of adjuvants, only very few, including AS04 (a combination of monophosphoryl lipid A (MPL) and alum) [7,8] or oil-in-water emulsions containing squalene (MF59 [9,10,11] and AS03 [12,13]) are approved today, mainly due to toxicity concerns since they often contain poorly defined fragments of bacteria or toxins with a low batch-to-batch reproducibility [14]. Although a few other adjuvants have been approved for specific vaccine formulations, alum is the only widely used adjuvant in commercial vaccines [15]. In 1926, Glenny et al. were the first to report the immune enhancement effect of insoluble aluminum potassium sulfate added to diphtheria toxoid [16]. Nowadays, the term “alum” describes adjuvant formulations based on aluminum hydroxide (Al(OH)_3_ and aluminum phosphate (Al(OH)_x_(PO_4_)_y_) that are commercially available or prepared by vaccine companies [17,18]. Alum is still the most widely used adjuvant today and typically generates Th2-polarized T cell responses, with a predominance of Th2-associated antibody subtypes [19]. A tremendous effort has been put on broadening the adjuvant repertoire to enable the fine-tuning of immune responses. While a Th2-skewed response is desired for the production of antibodies and anti-parasitic immune responses, a Th1 response is needed for the defense against intracellular or viral pathogens. Since the potent adjuvant action is often correlated with increased toxicity and reactogenicity, the benefits of adjuvant incorporation into a vaccine need to be balanced with the risk of adverse side effects. Therefore, minimizing toxicity still remains a major obstacle in adjuvant research and vaccine development. The development of vaccines that are able to potently activate innate immune responses without relying on live attenuated viruses is particularly relevant for immunocompromised individuals such as young children on the one hand, and the elderly population on the other hand [20,21,22].

Thus, a non-toxic, biocompatible, generally applicable potent adjuvant, ideally combined with an efficient intracellular delivery platform, is urgently needed to facilitate the use and future development of peptide-based vaccines.

In general, adjuvants can be divided into immune-potentiators/modulators that mainly activate the innate immune system or into delivery systems, which are particulate systems designed to improve antigen uptake and presentation [23]. By combining both types into a single platform, highly potent and safe adjuvants could be generated. Due to their potency to modulate the immune system and their additional stabilizing effects on the antigens, (bio)material-based vaccines are recently gaining increased attention [24,25,26,27]. Nanoparticles are of special interest since their size is similar to cellular components, potentially improving their uptake by antigen presenting cells (APCs), which qualifies them as a promising self-adjuvanting delivery system [28]. Silica particles have been widely used for drug delivery [29,30,31] and, recently, also for cancer vaccines [32,33,34] owing to their large surface area, large pore sizes, biodegradability and versatile surface chemistry [35,36]. In addition, silica is chemically and biologically quite inert and considered safe [37]. Nevertheless, it has been reported to be degraded in vitro as well as in vivo into non-toxic silicic acid that can be excreted through the kidneys [38,39,40,41]. In contrast, delivery vehicles based on polymers, synthetic or natural, often degrade into toxic products or tend to accumulate in the body [39,40,42,43]. 

Here, we describe a novel vaccine delivery and adjuvant platform employing biomimetic silica particles of different shapes (spheres, rods and sheets). These particles are formed by a biomimetic process taking advantage of three different peptides based on the silaffin R5 amino acid motif RRIL as well as by the parent peptide R5 [44]. All four silica-precipitating peptide have been linked to three different peptide antigens. The resulting antigen-loaded silica particles combine the advantages of biomaterial-based vaccines, such as being non-toxic and easy to generate, with intracellular uptake of silica particles.

## 2. Experimental Section

**Reagents.** Fmoc-preloaded resins were obtained from Rapp Polymere (Tübingen, Germany). Fmoc-protected amino acids were obtained from Iris Biotech (Marktredwitz, Germany). Boc-Lys(Fmoc)-OH was obtained from Orpegen Peptide Chemicals GmbH (Heidelberg, Germany). (1-[Bis(dimethylamino)methylene]-1H-1,2,3-triazolo-[4,5-b]-pyridinium 3-oxide hexafluorophosphate (HATU) was purchased from Novabiochem (Nottingham, UK).

Solvents were obtained from VWR International LLC (Vienna, Austria), Sigma-Aldrich by Merck GmbH (Vienna, Austria) and Carl Roth (Karlsruhe, Germany) in HPLC grade or peptide synthesis grade. All other chemicals used were obtained from Sigma-Aldrich (Taufkirchen, Germany).

**Water.** A Milli-Q Reference A+ water purification system by Merck GmbH (Vienna, Austria) was used for the purification of water deionized with a Professional G7895 Aqua Purificator by Miele GmbH (Salzburg, Austria).

**Animals.** Female BALB/c mice aged 6–8 weeks were purchased from Charles River (Sulzfeld, DE) and kept under conventional housing conditions. All experiments were approved by the Animal Experimentation Committee of the Medical University of Vienna, the University of Veterinary Medicine and the Austrian Federal Ministry of Science and Research (BMBWF-66.009/0277-V/3b/2019). All methods were performed according to their guidelines and regulations.

**Blood donors.** The peripheral blood cells were isolated from healthy individuals. The study had been approved by the ethics committee of the Medical University of Vienna (EK 1488/2017) and all subjects gave written informed consent.

**Peptide synthesis.** Peptides were synthesized manually or on a Liberty Blue microwave peptide synthesizer (CEM GmbH, Kamp-Lintfort, Germany) or on a Tribute peptide synthesizer (Gyros Protein Technologies, Inc., Manchester, UK) using Fmoc-protected amino acids. Tentagel PHB R resin preloaded with Fmoc-Leu for the CysR5, RRIL and RRLL peptides, Fmoc-Tyr for Bet v 1, Fmoc-Lys(Boc) for Phl p 5 and Fmoc-Cys for P467 at scales between 0.05 and 0.1 mmol was used. Amino acids were activated with HATU for arginine, leucine and lysine and HBTU for all other amino acids. Double couplings were performed for arginine and amino acids following arginine. 

The resin was swollen in DMF for at least 60 min. Then, the following steps were performed to extend the peptide by one amino acid. The resin was agitated on a wheel shaker during each deprotection, coupling and cleaving step. For the deprotection, DMF was removed and the resin was suspended in 20% piperidine in DMF. The solution was replaced after 3 min and the resin was incubated for another 7 min. Then, the resin was washed 5 times with DMF. For the coupling, the Fmoc-protected amino acid was first activated by dissolving 3.8 equivalents in a 0.5 M solution of HATU (3.5 equivalents) in DMF and adding 5 equivalents of DIPEA. After 3 min incubation, the solution was added to the resin and incubated for 30 min. Afterwards, the solution was removed, and the resin was washed 5 times with DMF. The peptide’s molecular weight was confirmed by performing a test cleavage and LC–MS analysis before continuing with the cleavage.

After the final deprotection, the peptide-loaded resin was washed 5 times with DMF, 5 times with DCM, and dried in the desiccator overnight. The resin was incubated in 40 mL/mmol cleavage solution (90% TFA, 5% TIS, 5% ddH_2_O or 90% TFA, 2.5% TIS, 2.5% ddH_2_O, 5% DMS for Met-containing peptides) for 3 h at room temperature. Subsequently, the peptides were precipitated with five times their volume in cold diethyl ether and after centrifugation (4300 rpm, 5 min, 4 °C) the supernatant was removed. The pellets were washed 3 times with diethyl ether and dried in an argon stream. Before purification, the pellets were dissolved in solvent A (0.1% TFA in ddH_2_O), flash frozen in liquid nitrogen and lyophilized to obtain the crude product.

**Preparative RP-HPLC.** Purification by reversed-phase high-performance liquid chromatography (RP-HPLC) was carried out on either a ProStar system by Varian, Inc., now Agilent Technologies (Santa Clara, CA, USA) or on a Waters AutoPurification HPLC–MS system (Saint-Quentin, France).

Peptides and peptide conjugates were purified using reversed-phase HPLC. After lyophilization, the crude peptide (up to 120 mg per batch) was dissolved in 10 mL guanidinium chloride buffer (6 M GuHCl, 50 mM Tris-HCl pH 7.5). The solution was filtered through a 20 µm syringe filter and loaded onto a Kromasil C4 prep column at 95% solvent A (0.1% TFA in ddH_2_O) and 5% solvent B (0.08% TFA in acetonitrile). Products were eluted using a suitable gradient of solvent B in solvent A with a flow rate of 20 mL/min. The obtained fractions were analyzed via direct injection into the MS system and those containing the desired product were pooled and lyophilized.

For the purification of silica-precipitating and antigen peptides a linear gradient from 5–55% solvent B in 40 min was used and for the purification of peptide conjugates, a gradient of 5–25% solvent B in 10 min, 25–45% solvent B in 30 min was employed. The obtained fractions were analyzed via direct injection into the MS system and those containing the desired product were pooled and lyophilized.

**Synthesis of heterodisulfide-bonded peptide conjugates.** Similar amounts of the silica-precipitating peptide and the peptide antigen were weighed in separately and transferred into the reaction vessel shortly before the guanidinium chloride buffer (6 M GuHCl, 50 mM Tris-HCl pH 7.5) was added. Prior to this, the guanidinium chloride buffer was degassed and flushed with argon. Disulfide-formation was initiated by addition of the buffer to the mixture of peptides and the solution was incubated for 24 h at room temperature. The formation of the heterodisulfide conjugate of the silica-precipitating peptide and peptide antigen was verified via LC–MS analysis and purification via preparative RP-HPLC was performed as described above.

**Analytical RP-HPLC.** For the final analysis via RP-HPLC, a small amount of the purified peptide was dissolved in solvent A, injected into a Dionex Ultimate 3000 HPLC system and eluated on an analytical Kromasil C4 RP-HPLC column using a gradient from 5–65% solvent B in solvent A over 30 min at a flow rate of 1 mL/min.

**Mass spectrometry.** Peptide identity and purity was investigated by ESI–MS on a Waters 3100 Mass Detector in positive ion mode.

**Synthesis of silica nanoparticles.** Silica-precipitating peptides were incubated at a concentration of 2 mg/mL in 50 mM potassium phosphate buffer at pH 7 for 24 h at room temperature. The concentration was determined by weighing the lyophilized peptide. A silicic acid solution was prepared by adding 40 µL TMOS to 960 µL 1 mM HCl, short vortexing, and incubation of the mixture for 4 min. A 10 µL portion of this silicic acid solution was added to a 90 µL aliquot of the peptide solution. The mixture was vortexed and incubated for 30 min at room temperature. Then, it was centrifuged (14,000 rpm, 5 min, room temperature) and the supernatant was removed. The particles were washed with ddH_2_O and dried at reduced pressure.

**Electron microscopy.** Suspensions of silica particles in ddH_2_O were prepared and 4 µL of the suspension was spotted onto 13 mm Nunc Thermanox cell culture coverslips from Thermo Fisher Scientific GmbH (Vienna, Austria) and dried on air. After sputter coating the samples with 10 nm of gold using an EM QSG 100 instrument by Leica Camera AG (Wetzlar, Germany), scanning electron microscopy was performed at a 5 kV acceleration voltage on a Supra 55 VP instrument by Carl Zeiss AG (Oberkochen, Germany) equipped with an EDX detector.

**Neutrophil isolation.** Neutrophils were isolated from heparinized peripheral blood by Ficoll–Paque gradients (Thermo Fisher Scientific GmbH, Vienna, Austria), dextran sedimentation (4% Dextran T-500, Carl Roth, Karlsruhe, Germany), and osmotic lysis of remaining erythrocytes as described previously [45].

**Quantification of extracellular DNA:** A total of 2 × 10^5^ neutrophils in 200 μL were seeded into black flat bottom 96-well plates (Thermo Fisher) in HBSS medium containing 25 ng/mL GM-CSF and 5 μM of the cell-impermeable DNA-dye SYTOX orange (Thermo Fisher). After 30 min of priming, cells were stimulated in triplicates with ionomycin, alum (Alu-Gel-S) or test samples, at the indicated concentrations. Fluorescence was measured at 575 nm with a TECAN Infinite M1000 fluorescence reader (Tecan, Zürich, CH, Switzerland) every 2 min for up to 3 h.

For the inhibition experiment, the neutrophils were pre-incubated in the presence of 25 ng/mL GM-CSF for 30 min with the phagocytosis inhibitor cytochalasin D (10 µg/mL).

**Stimulation of bone marrow-derived dendritic cells.** Bone marrow precursor cells were isolated from murine femurs and tibias of naïve female BALB/c mice and cultured in complete medium (RPMI-1640 supplemented with 10% heat-inactivated FCS, 2 mM L-glutamine, 2 mM mercaptoethanol, 100 μg/mL gentamicin; Sigma-Aldrich) supplemented with GM-CSF (20 ng/mL; Peprotech, London, UK) as described previously [46]. On day 8, bone marrow-derived dendritic cells (BMDC) (1 × 10^6^/mL) were incubated with medium only, with 50 µg/mL or 100 µg/mL of the examined peptides, peptide conjugates and silica particles, LPS (1 μg/mL) or Pam3 (1 µg/mL) for 24 h. The BMDC cultures were kept in the incubator at 37 °C and 5% CO_2_. Supernatants were collected and stored at −20 °C until use. Detection of cytokines IL-6, and TNF-α in supernatants was carried out by ELISA (Ready-Set-Go Kit, eBioscience, USA) according to the manufacturer’s instructions using SparkControl 10M plate reader (Tecan GmbH, Salzburg, Austria).

**Thermogravimetric analysis.** The measurements were performed using a Netzsch STA 449-F3 Jupiter analyzer, from room temperature to 800 °C with a heating rate of 10 °C/min. An air flow of 20 mL/min was used as carrier gas with an additional protective N_2_ flow of 20 mL/min.

**Nitrogen physisorption analysis.** Nitrogen physisorption analyses at −196 °C were performed with a Quantachrome iQ3 instrument (Anton Parr, Boynton Beach, FL, USA). Prior to the measurements, the samples were outgassed under vacuum for 24 h at 35 °C. The specific surface area (S_BET_) was obtained using the BET equation in the range of 0.1–0.2 P/P_0_. The total pore volume was determined at P/P_0_ = 0.95 according to the Gurvitch rule, and the pore size analysis was calculated using the metastable adsorption NLDFT model (adsorption branch), assuming a cylindrical pore geometry and a silica surface. Calculations were performed with the ASiQwin software v.5.0 provided by Quantachrome.

**Wide-angle powder XRD analysis.** The wide-angle diffractograms of silica particles were recorded on a PANalytical Empyrean diffractometer (Malvern PANalytical, United Kingdom) in reflection geometry (Bragg−Brentano HD) using Cu Kα_1 + 2_ radiation operated at a voltage of 45 kV, a tube current of 40 mA, and with a fixed divergence slit of 0.05 mm. Measurements were performed in continuous mode with a step size 2 theta of 0.013° and a time per step of 200 s.

**Statistical analysis.** Data were statistically analyzed by GraphPad Prism software (Graph Pad Software, USA) utilizing unpaired Student’s *t*-test and two-way ANOVA. All data are shown as the mean ± standard error of the mean (StEM) and differences were considered significant at *p* < 0.05.

## 3. Results

### 3.1. Synthesis of Silica-Precipitating Peptides and Morphological Evaluation of Silica Particles Resulting from Biomimetic Precipitation

We began our studies by first synthesizing the silica-precipitating peptides CysR5, RRIL-1, RRIL-2 and RRLL (Table 1), which have been previously described by us and others as effective silica particle forming entities [47,48,49,50,51,52]. All of them were obtained with isolated yields of 61–72% (based on amount of crude peptide) and in high purity of >95% (Figures S1–S4). To characterize the biomimetic silica particles resulting from these peptides, they were incubated in 50 mM potassium phosphate buffer at pH 7 at a concentration of 2 mg/mL. The silica precipitation was initiated upon addition of freshly generated silicic acid (from hydrolysis of tetramethylorthosilicate-TMOS). After incubation at room temperature and centrifugation, silica particles were collected by centrifugation and dried under reduced pressure. To analyze the amount of peptide that is entrapped within the silica particles, aliquots were taken before and after the silica precipitation and run on RP-HPLC. Peptide incorporation was determined by integration of RP-HPLC peak areas. Scanning electron microscopy (SEM) was employed to evaluate size and morphology of the resulting particles. In addition, elemental composition of the particles was analyzed by energy dispersive X-ray (EDX) spectroscopy (Figure S5). All particles contained Si, O, C, and N as well as Au as expected for peptide containing silica particles that were sputter-coated with a thin layer of gold during SEM sample preparation.

**Figure 1:**
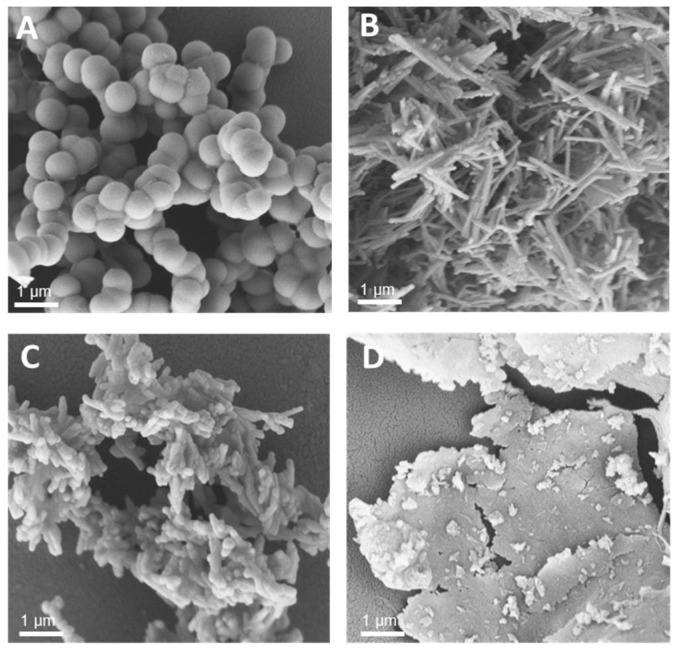
Scanning electron microscopy (SEM) images of silica particles generated from CysR5 1′ (**A**), RRIL-1 2′ (**B**), RRIL-2 3′ (**C**), and RRLL 4′ (**D**). Scale bar represents 1 µm.

Under the above-mentioned conditions, particles generated by precipitation with CysR5 (**1′**) showed spherical particles with an average diameter of 800 ± 45 nm as determined by measurements of 20 individual particles (*n* = 20), RRIL-1-based particles (**2′**) gave rod-like structures with individual rods measuring up to 1 µm in length and 104 ± 11 nm (*n* = 10) in diameter while RRIL-2-based particles (**3′**) were shaped as shorter, thicker rods with an average length of 500 nm and 162 ± 19 nm (*n* = 10) in diameter. RRLL-based particles (**4′**) showed a sheet-like morphology that carried additional stick-like structures on top as can be seen in Figure 1. In the subsequent sections, silica particles are labeled as **x’** and calcinated silica particles are labeled as **x’’**, with **x** being a number. All structures were in accordance with earlier findings of Lechner et al. and Kamalov et al. [49,53]. Having all these particles in hand, we first tested their immunogenicity by analyzing their ability to induce the formation of neutrophil extracellular traps (NETs) from human neutrophils.

### 3.2. Silaffin-Derived Silica Particles Induce NET Release

Not only particle size but also shape, surface charge and the protein corona greatly influence the immunological response to such particles. To elucidate the immunogenicity of the obtained silica particles based on NET formation, freshly isolated human neutrophils were seeded in a 96-well plate, primed with GM-GSF, incubated with medium or stimulated with alum (as established adjuvant), ionomycin (as control immune-stimulator) and the four different silica particles. NETs are web-like structures composed of nuclear and granular proteins that are embedded in a scaffold of decondensed chromatin [54]. It has been demonstrated, that NETs are involved in the adjuvant effect of alum in mice [55,56]. Although the mechanisms underlying alum’s adjuvant effect in humans remain largely unclear, it has been shown that injection of alum induces neutrophil infiltration and NET formation in human tissue [57]. Therefore, NETs may contribute to the alum-induced immune response to vaccines. To analyze the kinetics and extent of NET release, an established assay based on the addition of SYTOX™ orange, a cell membrane-impermeable DNA-dye, to neutrophils was used [57]. SYTOX™ orange binds selectively to extracellular DNA and the resulting fluorescence signal can be measured.

**Figure 2:**
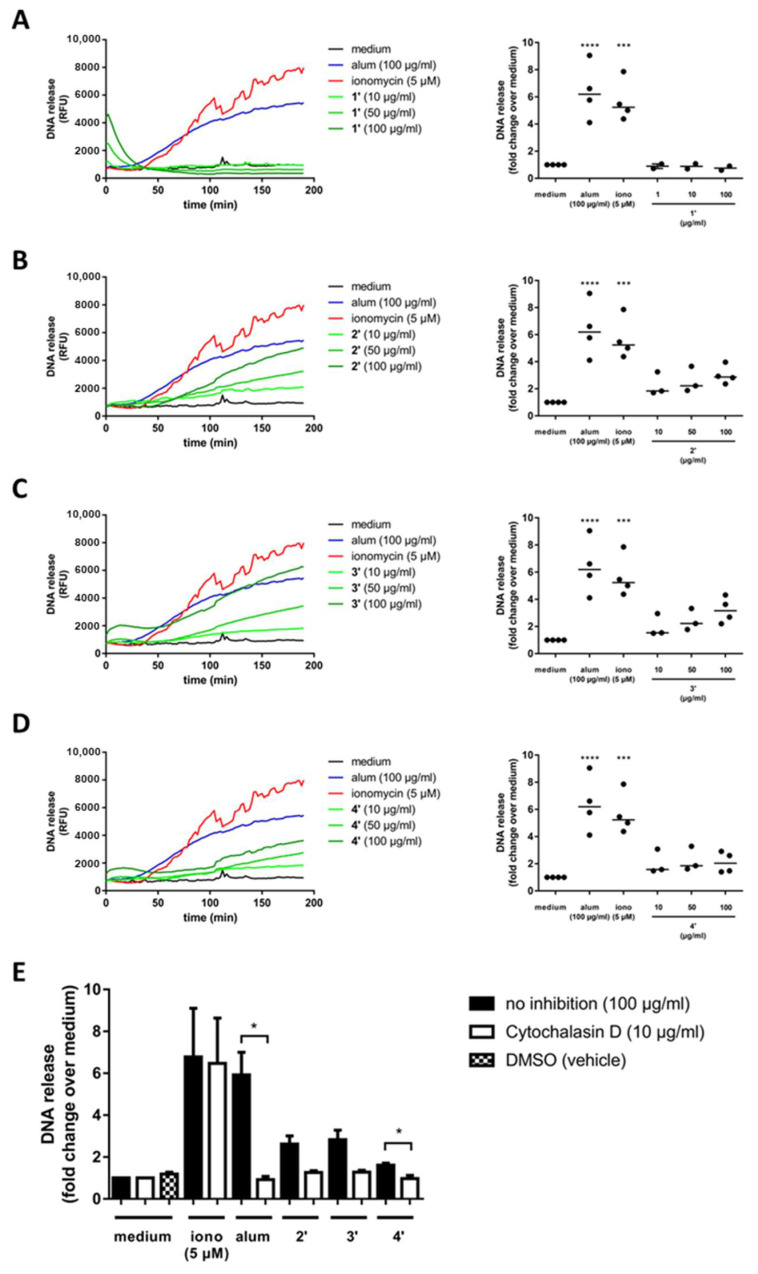
Silica particles trigger NET formation. The kinetics of alum, ionomycin and CysR5 **1′** (**A**), RRIL-1 **2′** (**B**), RRIL-2 **3′** (**C**), and RRLL **4′** (**D**) silica particle-induced release of DNA were measured by fluorescence of SYTOX orange bound to extracellular DNA in plate reader assays. One representative experiment using neutrophils from different donors (AUC, area under the curve; RFU, relative fluorescence units; horizontal bar, median). Alum, ionomycin and silica particle-induced NET formation in neutrophils which had been pre-incubated for 30 min with the phagocytosis inhibitor cytochalasin D (**E**). Differences were considered statistically significant for values of *p* ≤ 0.05. Note: *p* ≤ 0.05 (*), *p* ≤ 0.001 (***), *p* ≤ 0.0001 (****).

Alum and ionomycin both trigger a concentration-dependent NET release after 30 min of stimulation as described previously [57,58]. In contrast, spherical CysR5-based particles **1′** reach the highest NET release right at the beginning of the measurement period, which then rapidly decreases to baseline levels within 40 min (Figure 2A–D). Such an immediate NET release indicates a necrotic event, which could suggest a cytotoxic effect. In contrast, rod-like RRIL-1 particles **2′** induce concentration-dependent NET release after 50 min, which is slightly slower compared to alum or ionomycin. However, at the end of the measurement period, the highest concentration of RRIL-1 particles **2′** (100 µg/mL) tested here leads to a similar level of NET release as alum at the same concentration. Although RRIL-2 particles **3′** resemble RRIL-1 particles **2′** in terms of release kinetics, they lead to an even higher NET release at the end of the measurement period after 180 min. RRLL particles **4′** trigger NET release with a delay only after 60 min and reach approximately half the extent of NET release compared to alum. However, due to the different release kinetics of alum, ionomycin and the silica particles, the DNA release calculated as area under the curve (AUC) over time might be slightly misleading, because RRIL-1 **2′** and RRIL-2 particles **3′** induce NET release 20 min later than alum and have not yet reached a plateau at the end of the measurement as opposed to alum. Nevertheless, these results show that RRIL-1 **2′** and RRIL-2 particles **3′** can stimulate NET release in human neutrophils to a similar extent as the well-established adjuvant alum.

To elucidate the silica particle-induced cellular mechanisms causing NET formation, cells were also preincubated in the presence of GM-CSF for 30 min and the Ca^2+^-dependent phagocytosis inhibitor cytochalasin D (Figure 2E). Addition of cytochalasin D clearly reduced NET release in alum and silica particle-stimulated neutrophils, which indicates that silica particle-induced NET formation might follow similar cellular mechanisms as proposed for alum [57].

The observed release of extracellular DNA in human neutrophils elicited by silica particles prompted us to investigate NET formation in vitro by fluorescence microscopy as an alternative route to quantitate this effect [57]. To that end, we also generated calcinated silica particles from the regular silica particles to gain insights about the role of the precipitating peptides on particle as well as on adjuvant properties. Precipitating peptides within the silica particles were removed by calcination at 100 °C for 12 h followed by 5 h at 540 °C. This way, we were able to individually test the peptides alone, peptides in silica particles and calcinated silica particles. Freshly isolated neutrophils were seeded on glass coverslips, primed with GM-CSF, incubated with medium or stimulated with ionomycin, CysR5 **1** as well as RRIL-2 peptides **3**, silica particles and calcinated silica particles and evaluated for NET formation by fluorescence microscopy. The medium control (Figure S6A) displayed lobular nuclei. NET release was induced in ionomycin-stimulated neutrophils (Figure S6B) that showed disintegrated nuclei and filamentous, web-like extracellular DNA. The co-localization with citrullinated histone H3 (staining with red, Alexa Fluor 594 used as marker for NET release), corroborate that those DNA-webs are indeed NETs. In (Figure S6C) and (Figure S6D) cells treated with RRIL-2 **3** and CysR5 peptide **1** are depicted, showing almost no signs of NET release when compared to the positive control. Stimulation with silica particles as seen in (Figure S6E) and (Figure S6F) resulted in typical agglomerates of cells. However, only a small portion of cells showed NET release. In addition, the cells were unevenly distributed, as they tend to cluster in areas where the particles are present. The stimulation with calcinated silica particles (Figure S6G) and (Figure S6H) resulted in a similar image compared to the silica particles. Nevertheless, in comparison, the proportion of citrullinated histone H3 positive cells was lower.

**Figure 3:**
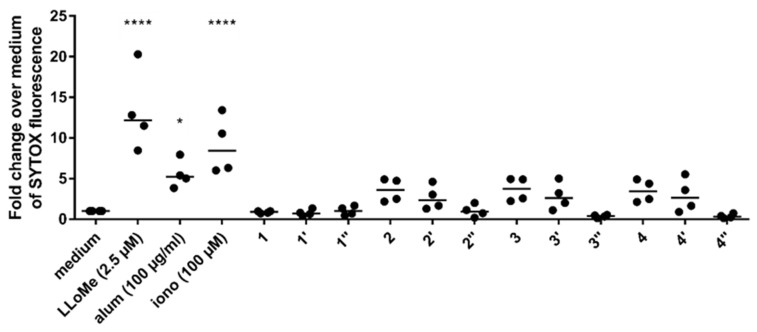
Peptides and silica particles trigger NET formation. The release of DNA was measured by fluorescence of SYTOX™ orange bound to extracellular DNA in plate reader assays in response to treatment with peptides (**1**, **2**, **3** and **4**) silica particles (**1′**, **2′**, **3′** and **4′**) and calcinated silica particles (**1″**, **2″**, **3″** and **4″**). l-leucyl-l-leucine methyl ester hydrochloride (LLoMe), alum and ionomycin (iono) were used as positive controls. One representative experiment using neutrophils from different donors (horizontal bar, median). Differences were considered statistically significant for values of *p* ≤ 0.05. Note: *p* ≤ 0.05 (*), *p* ≤ 0.0001 (****).

In general, peptide samples (**2**, **3** and **4**) induced the highest level of NET release, followed by silica particles (**2′**, **3′** and **4′**) and calcinated silica samples (**1″**, **2″**, **3″** and **4″**) that did not elicit any observable release of extracellular DNA as can be seen in Figure 3. Stimulation of neutrophils with RRIL-2-based compounds resulted in the most potent NET formation although RRLL and RRIL-1 induced similar levels. In contrast, no release of extracellular DNA was observed for CysR5-based compounds (**1**, **1′** and **1″**). However, no significant effect was observed for any of the tested compounds. This might be due to the small number of donors (*n* = 4) in this study and high variance of the data resulting from highly individual immune responses. Therefore, additional studies with a bigger sample size need to be performed to test this trend.

### 3.3. Silica Particles Loaded with Peptide–Antigen Conjugates

**Figure 4:**
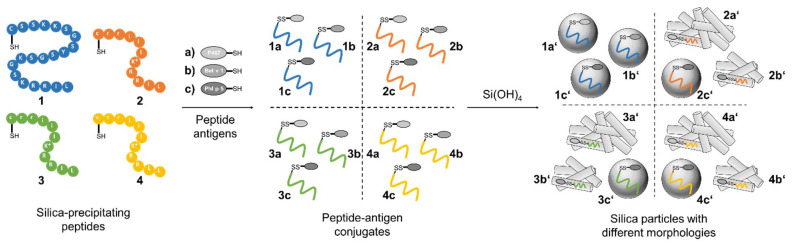
Conjugation of four silica-precipitating peptides (CysR5 **1**, RRIL-1 **2**, RRIL-2 **3** and RRLL **4**) to three peptide antigens (P467 **a**, Bet v 1 **b**, and Phl p 5 **c**) via disulfide bond formation. Addition of silicic acid leads to the formation of 12 different peptide–antigen silica particles with different morphologies including spheres, rods, and interwoven networks. CysR5-related structures are displayed in blue, RRIL-1-related structures in orange, RRIL-2-related structures in green and RRLL-related structures in yellow.

To investigate the adjuvant effect of the different silica particles in combination with established peptide antigens, the major white birch pollen allergen Bet v 1 [59,60,61], the major timothy grass pollen allergen Phl p 5 [61,62] and the hybrid peptide P467 [63,64,65], derived from the extracellular domain of the tumor-associated antigen Her-2/neu, were conjugated to each silica-precipitating peptide through the formation of a disulfide-linkage between the sulfhydryl moieties on the side chain of the cysteine residues as illustrated in Figure 4. The formation of the desired disulfide bridges to provide peptide conjugates was achieved by incubating the reduced silica-precipitating peptides 1–4 as well as the reduced peptide antigens in guanidinium buffer (6 M guanidinium hydrochloride, 50 mM Tris, pH 7.5) overnight. During air oxidation, not only the desired disulfide bond between silica-precipitating peptide and antigen was formed, but also the respective peptide–peptide and antigen–antigen disulfide bridges occurred, which reduces the yield. Nevertheless, to reduce synthetic complications and provide a simple preparation route for silaffin peptide–antigen conjugates, no protecting groups were employed. All conjugates were obtained in isolated yields of 24–82% and in high purity (Figures S7–S21) and used for silica particle generation. All conjugates were tested towards their ability to induce silica precipitation form silicic acid under biomimetic conditions. SEM was employed to evaluate the changes in silica particle size and morphology upon conjugation of antigens. As expected, no silica formation was observed for the peptide antigens alone.

Conjugation of three established antigens to CysR5 resulted in particles that retained their spherical morphology and size in comparison to particles made from CysR5 alone. The obtained average diameters are 796 ± 91 nm (*n* = 10) for CysR5-P467 particles **1a′**, 789 ± 52 nm for CysR5-Bet v 1 particles **1b′** (*n* = 10) and 840 ± 70 nm (*n* = 10) for CysR5-Phl p 5 particles **1c′**. This was expected as we have previously demonstrated that even much larger cargo molecules such as the proteins GFP and thioredoxin do not impact R5-controlled silica precipitation. [52] The morphology of rod-like RRIL-1 particles changed significantly upon conjugation of the model antigens. Conjugation of Bet v 1 to RRIL-1 resulted in a network of rod-like particles **2b′** that were similar to the morphology of RRIL-1 particles, but shorter and with a smaller diameter of 87 ± 15 nm (*n* = 10). The RRIL-1-Phl p 5 conjugate led to irregular, spherical particles **2c′** with an average diameter of 370 ± 68 nm (*n* = 10) and the RRIL-1-P467 conjugate also formed a dense network of thin, rod-like particles **2a′** with a diameter of 40 ± 6 nm (*n* = 10). A dramatic change in morphology could also be observed for RRIL-2 particles that normally present themselves as stick-like structures. The particles **3b′** made from the RRIL-2-Bet v 1 conjugate with a diameter of 72 ± 14 nm (*n* = 10) resemble the particles made from the respective RRIL-1 conjugate since they both generated a network of short and small rod-like particles. This also applies to the morphology of the particles **3a′** generated from RRIL-2-P467 that produced an interwoven network of thin rods with a diameter of 24 ± 4 nm (*n* = 10). This observation holds also true for RRIL-2-Phl p 5 particles **3c′** that were obtained as spherical particles with a diameter of 485 ± 44 nm (*n* = 10). As already observed for RRIL-1 and RRIL-2, the sheet-like morphology of RRLL particles changes to a network of rod-like particles with a diameter of 127 ± 19 nm (*n* = 10) for Bet v 1 **4b′** and 43 ± 4 nm (*n* = 10) for P467 **4a′** and spherical particles with a diameter of 720 ± 59 nm (*n* = 10) that resemble CysR5 particles for RRLL-Phl p 5 **4c′**. These differences in morphologies clearly indicate that the shorter RRIL-based silaffin peptides are severely influenced by the covalent attachment of cargo (peptides). This might be a simple effect of the size of the silica-precipitating peptides covalently linked to the model antigens or can also be affected by changes in charge patterns, which together interfere with pre-assembly of peptide templates. CysR5 comprises 20 amino acids and seems to be the driving force behind the assembly of CysR5-antigen conjugates although Phl p 5, consisting of 22 amino acids, and the 49 mer P467 are slightly longer. However, the RRIL/RRLL peptides comprise only 11 amino acids and seem to adapt their assembly behavior depending on the antigen since similar morphologies were observed for the antigen conjugates, e.g., a network of rod-like particles for Bet v 1 and P467 and spherical particles for Phl p 5.

In addition, we determined how much of the peptide conjugate was incorporated into the silica particles by analyzing aliquots on HPLC before and after silica precipitation. The results are displayed in Table 2.

**Figure 5:**
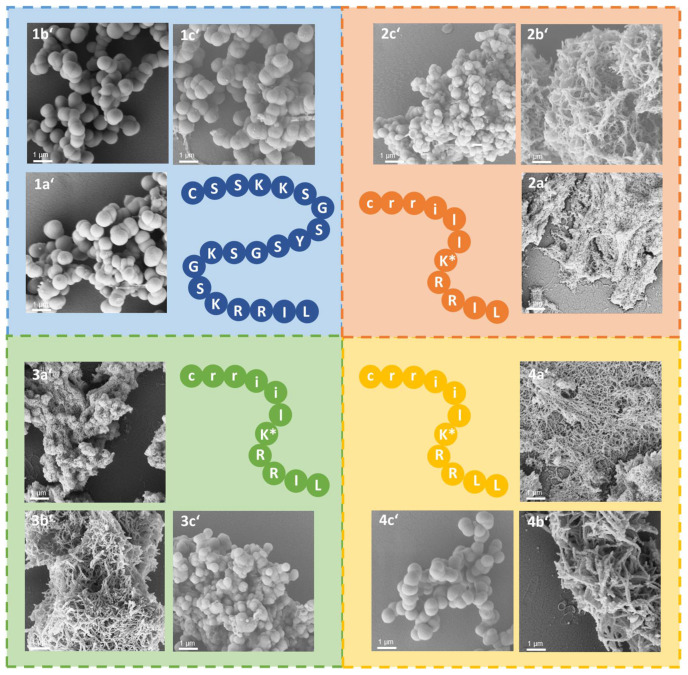
SEM images of silica particles generated from CysR5, RRIL-1 **2**, RRIL-2 **3** and RRLL **4** conjugated to P467 **a**, Bet v 1 **b** and Phl p 5 **c**. CysR5-related structures are displayed in blue, RRIL-1-related structures in orange, RRIL-2-related structures in green and RRLL-related structures in yellow. Scale bar represents 1 µm. * Indicates branching via the lysine (K) sidechain.

While CysR5-based conjugates achieve incorporation efficiencies between 30–40%, RRIL-2 conjugates are incorporated with 85–90% efficiency. Although CysR5 conjugates are not encapsulated as efficiently as RRIL-2 conjugates, the spherical shape of their respective silica particles is reproducible independent of the cargo [51,53]. In contrast, the morphology of silica particles made from RRIL-2 conjugates is dependent of the cargo as can be seen in Figure 5. 

Furthermore, we analyzed how much of the CysR5-Phl p 5 peptide–antigen conjugate was released from the respective silica particles during incubation at pH 4 and pH 7 to assess stability under different relevant conditions (e.g., in media, cytosol and lysosomes). The silica particles produced from CysR5-Phl p 5 peptide–antigen conjugates were incubated in 50 mM potassium phosphate buffer at pH 4 and pH 7 for 11 h to monitor the release of the conjugate from the particles. By comparing the area under the curve (AUC) of the peptide conjugate at different time points with the AUC of a standard of known concentration, the release was determined as displayed in Figure S22.

At pH 4 approximately 20% of the conjugate were released, whereas only about 8% were released from particles at pH 7. Electrospray ionization-mass spectrometry (ESI–MS) analysis confirmed that the conjugate was intact after 11 h incubation at pH 4. Additionally, no morphological changes to the particles due to the incubation could be observed via SEM as shown in Figure S23. This indicates that release of the conjugate is not induced through the degradation of the silica matrix. Therefore, ionic interactions of the conjugate with the silica seem to be responsible for the partial release that is induced via change in pH. The negatively charged silica surface [66] becomes neutral at low pH which results in weakened interactions with the cationic conjugate peptide, ultimately leading to its release. This is in accordance with literature as described by Bialas et al. [67].

### 3.4. Biophysical Characterization of Silica-Precipitating Peptides

The ζ potential of CysR5 **1′**, RRIL-1 **2′**, RRIL-2 **3′** and RRLL **4′** silica particles before and after calcination was determined over a range of pH 4–8 (Figure S24) Before calcination, the peptide-containing silica particles displayed a positive zeta potential of 5 to 15 mV at pH 7. During calcination, the positively charged silica-precipitating peptides are removed via thermal decomposition. Therefore, the zeta potential of all the resulting pure silica particles decreased to approximately −42 to −30 mV at pH 7, which is in accordance with literature values for silica particles [68].

**Figure 6:**
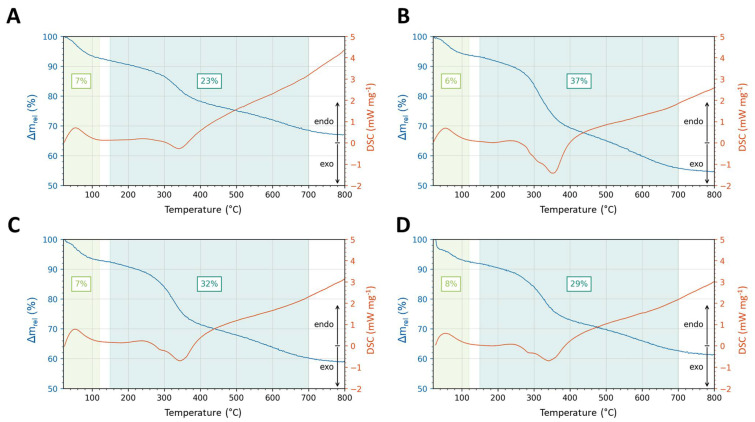
Thermogravimetric and differential thermal analysis of CysR5 **1′** (**A**), RRIL-1 **2′** (**B**), RRIL-2 **3′** (**C**), and RRLL **4′** (**D**) silica particles.

To determine the organic content and chemical stability of as-made CysR5 **1′**, RRIL-1 **2′**, RRIL-2 **3′** and RRLL **4′** silica particles, thermogravimetric analysis (TGA) was carried out monitoring the weight loss as a function of increasing temperature up to 800 °C under air flow. As shown in Figure 6, the TGA curves of the samples all show a first weight loss up to 120 °C ranging from 6 to 8%, due to the evaporation of physiosorbed water molecules. The percentage of weight loss at temperatures between 150–700 °C was 23% for CysR5 **1′**, 37% for RRIL-1 **2′**, 32% for RRIL-2 **3′** and 29% for RRLL **4′** particles and corresponds mainly to the loss of peptide material contained within the silica particles (with a contribution of water from the silanol condensation process). The thermal decomposition of the organic parts follows a stepwise process, associated with successive exothermic differential thermal analysis (DTA) effects, as can be seen in Figure 6. Typically, the thermal unfolding of peptides starts at 40 °C; however, the silica matrix seems to act as a stabilizer and increases the thermal resistance of the peptide, as described in the literature [69].

**Figure 7:**
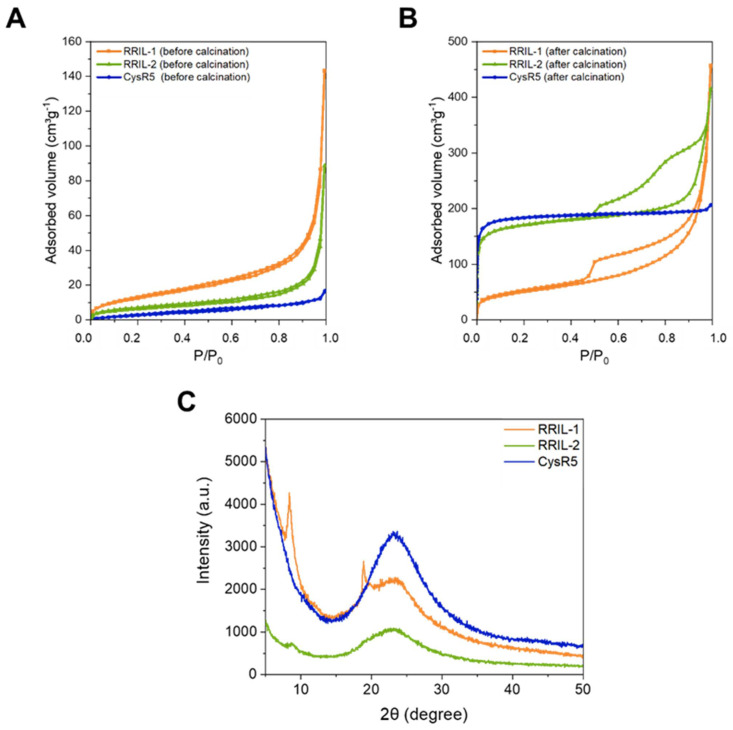
Nitrogen physisorption isotherms measured at −196 °C of CysR5 (blue), RRIL-1 (orange) and RRIL-2 (green) silica particles before (**A**) and after (**B**) calcination. Wide-angle powder XRD patterns (**C**) of CysR5 **1′** (blue), RRIL-1 **2′** (orange), and RRIL-2 **3′** (green) silica particles.

To gain insights about the porosity of the of CysR5, RRIL-1 and RRIL-2 silica-based particles before and after calcination, nitrogen physisorption analysis at -196 °C was performed. The physisorption isotherms of CysR5 **1′** (blue), RRIL-1 **2′** (orange) and RRIL-2 **3′** (green) silica particles are shown in Figure 7 before (A) and after (B) calcination. The physicochemical parameters derived from gas adsorption are compiled in Table 3. Before calcination, the samples show low porosity as reflected by isotherm curves indicating mostly non-porous materials. One can notice an increase in the adsorption volume at high relative pressure, which could be attributed to external surface area originating from some interparticle pores/voids, which is typical for colloidal silica materials [70,71]. Only sample RRIL-1 **2′** shows a non-negligible (internal) porosity before calcination, with a slightly increased adsorbed volume at low pressure (leading to a modest surface area of 48 m^2^/g), the origin of which remains unclear at the moment.

As expected, a pronounced increase in specific surface area and pore volume is observed after calcination for all silica materials. The increase in porosity is caused by removal of the silica-precipitating peptides upon calcination. This corroborates the encapsulation of the peptides within the silica particles and their role as a biological porogen. This effect was most pronounced for CysR5 **1′** and RRIL-2 **3′** silica particles with a substantial increase in surface area from 13 to 727 m^2^ g^−1^ for CysR5 and 22 to 650 m^2^ g^−1^ for RRIL-2 silica particles. On the other hand, for RRIL-1 silica particles **2′** only a moderate increase in surface area from 48 to 180 m^2^ g^−1^ was observed. As can be seen from the wide-angle powder X-ray diffraction (XRD) spectrum in Figure 7C, solely the RRIL-1 peptide **2** shows two distinct peaks that are indicative of crystals that grow not only within the particles but are also located on the outside. This finding indicates that the RRIL-1 peptide **2** has a different mode of self-assembly compared to CysR5 **1** and RRIL-2 **3** that results in a smaller internal surface area upon silica precipitation.

**Figure 8:**
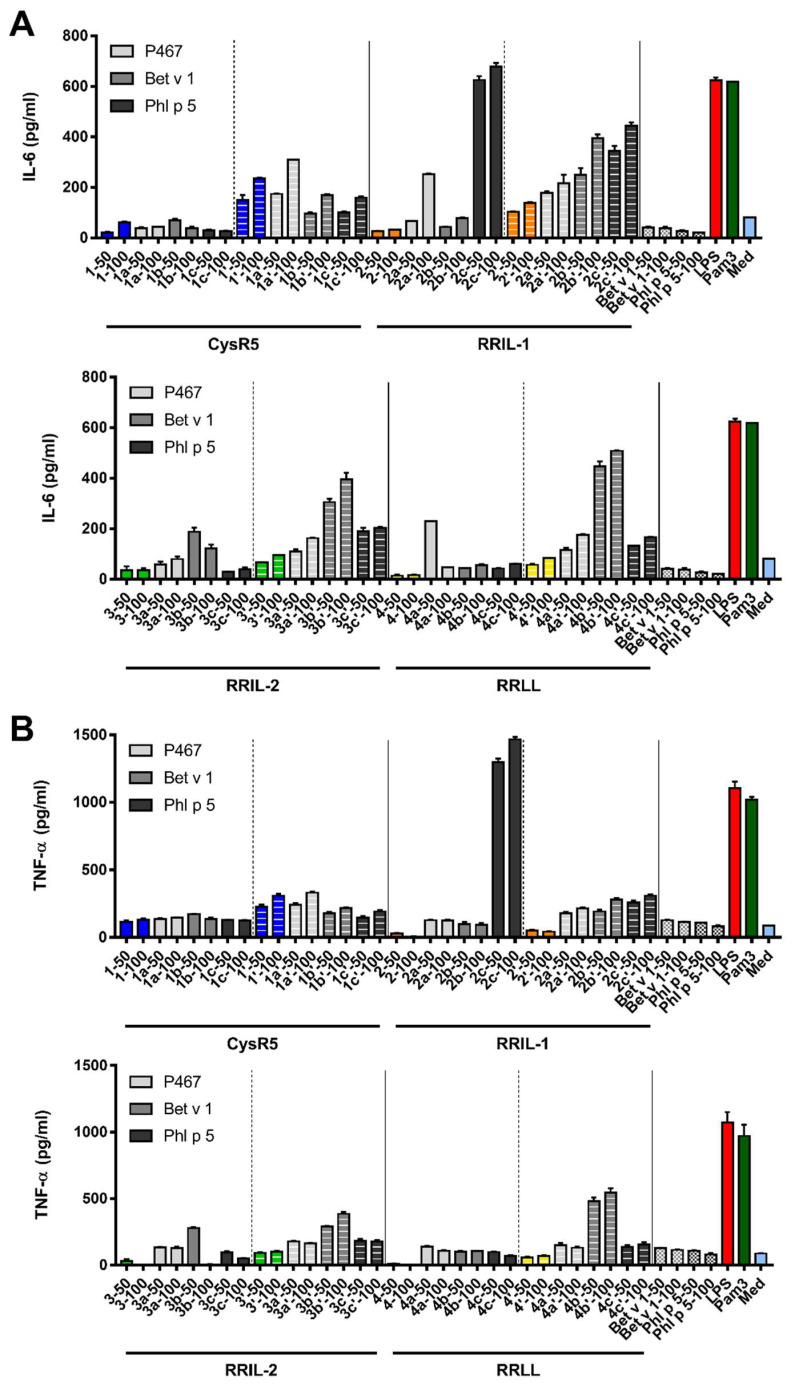
IL-6 (**A**) and TNF-α (**B**) secretion by murine bone marrow-derived dendritic cells in response to 50 µg and 100 µg of the silica-precipitating peptides, peptide–antigen conjugates and to silica particles produced from the conjugates was employed to assess their immunostimulatory effects. CysR5-related structures are displayed in blue, RRIL-1-related structures in orange, RRIL-2-related structures in green and RRLL-related structures in yellow. LPS (red) and PAM3 (dark green) were used as positive controls and medium (light blue) as negative control.

After calcination, Figure 8B shows that the nitrogen adsorption-desorption isotherm of CysR5 **1″** is a typical Type I isotherm according to the IPUAC classification [72], indicating that the product has a microporous structure. On the other hand, the isotherm corresponding to calcined RRIL-2 silica particles **3″** displays hybrid behavior with both Type I and Type IV characteristics and featuring a wide H2b/H4 type hysteresis loop with two desorption steps that could suggest a complex hierarchical pore structure with both internal micropores and different sets of larger (meso)pores [73,74]. 

Calcined RRIL-1 silica particles **2″** display a mixed Type II/IV isotherm with a very wide capillary condensation step and H3-type hysteresis loop characteristic of a wide distribution of mesopores and possibly macropores. The hysteresis loop closes through a cavitation effect suggesting that access to the large (interstitial) mesoporous or macroporous voids is controlled by smaller internal pores/entrances (<4–5 nm) [75,76]. Pore size distributions were calculated from the isotherms of the calcined materials using the NLDFT method (similarly, NLPSDs obtained for as-made samples are shown for comparison) [77,78]. As can be seen from Figure S25, the wide PSD of calcined RRIL-1 silica **2″** is centered around 6–7 nm, but it spreads towards large pore sizes as well. In contrast, calcined RRIL-2 **3″** and CysR5 **1″** samples demonstrate a narrow pore size distribution in the micropore range centered at 1.3 and 1.1 nm, respectively. This marked difference in pore size can also explain the observed difference in specific surface area between RRIL-1 silica **2″** and the other samples.

Figure 7C shows wide-angle powder X-ray diffraction (XRD) patterns for CysR5 **1′**, RRIL-1 **2′** and RRIL-2 **3′** silica samples. While CysR5 **1′** and RRIL-2 **3′** silica particles essentially displayed the characteristic halo of amorphous materials, RRIL-1 silica particles **2′** showed two distinct reflections at 8° and 19° (2 theta) that indicate a polycrystalline structure. This could be explained by the presence of the silica-precipitating RRIL-1 peptide **2** that would not only be confined inside the silica particles as a kind of pore template, but also located on the outside where larger crystals can grow [79].

### 3.5. Activation of Murine Bone Marrow-Derived Dendritic Cells by Silica Particles Generated from Peptide–Antigen Conjugates

Having all the different sample types in hand, the immunostimulatory effects of silica-precipitating peptides, peptide–antigen conjugates and silica particles made from silica-precipitating peptides and respective conjugates on murine bone marrow-derived dendritic cells (BMDCs) were elucidated by analyzing cytokine secretion in response to exposure to peptides and silica particles. To this end, bone marrow-derived dendritic cells were cultured in the presence of peptides and silica particles (see SI) [46]. LPS and PAM3 were used as positive controls and medium as negative control. The concentrations of a set of cytokines indicating immune stimulation (IL-6, IL-12p70, IFN-γ and TNF-α) in the culture supernatant were measured by ELISA. 

All silica particles activated the BMDCs to produce the pro-inflammatory cytokines IL-6 and TNF-α in a concentration-dependent manner as can be seen in Figure 6. However, no induction of IL-12p70 and IFN-γ was observed which points towards a Th2 skewed immune response [80,81]. Furthermore, silica particles made from CysR5 **1**, RRIL-1 **2**, RRIL-2 **3** and RRLL **4** were also able to induce IL-6 secretion. Here, CysR5 silica particles **1′** turned out to be remarkably potent and they also elicited a high level of TNF-α secretion. Not only the silica particles but also the peptide conjugates by themselves were able to induce IL-6, and to a lesser extent TNF-α secretion. Especially RRIL-1 and RRIL-2 peptide conjugates produced a robust IL-6 secretion, whereas CysR5 peptide conjugates stimulated a high TNF-α production. CysR5 **1** was the only silica-precipitating peptide that was able to stimulate IL-6 secretion although only at a very low level. In addition, it was the only silica-precipitating peptide to elicit a moderate level of TNF-α secretion.

## 4. Discussion

The aim of this study was to investigate immunomodulatory properties of silaffin and RRIL peptide-derived silica particles with varying morphologies and to evaluate their potential as immune cell stimulators. In addition, a first analysis of their biophysical characteristics was performed. To the best of our knowledge, this is the first study to elucidate the immunostimulatory effects of silaffin-derived silica particles, which combine the advantages of common silica particles with biomimetic production and control over morphology in the context of immune stimulation and potential use of such particles as carrier for subunit vaccines.

Silica particles with different morphologies and their respective peptide precursors were able to induce NET release in primary human blood-derived neutrophils. Based on the observation that the positively charged silica-precipitating RRIL peptides enhanced NET formation in contrast to the negatively charged calcinated silica particles, which abolished NET release independent of their shape (see Figure 3), we suggest a charge-dependent model of NET induction similar to the mechanism of cathelicidin LL-37 and alum as previously reported by Reithofer et al. [57]. In contrast to literature, we did not find significant changes in the ability to induce NETs based on different particle morphologies. 

However, not only charge, but also hydrophobicity seems to play a central role in the induction of NET release. In contrast to the RRIL peptides that have a substantial proportion of hydrophobic residues (>45%), in addition to their net charge of +5 under physiological conditions at pH 7.0, the CysR5 **1** peptide with a net charge of +6 lacks those hydrophobic parts (Table 1 and Table S1). The human antimicrobial peptide LL-37, a member of the α-cathelicidins, shares the cationic character and high content of hydrophobic residues (35%) with the RRIL peptides. Multiple studies [54,82,83,84] have demonstrated that LL-37 facilitates NET release in human neutrophils and also induces NET release from neutrophils in vivo in a CLP septic model of mice. Neumann et al. reported that LL-37-induced NETosis is related to the hydrophobic character of the peptide which is in accordance with other studies that show that the hydrophobicity of LL-37 is involved in the binding and disruption of bacterial membranes [85].

Therefore, we suggest that the positive charge and hydrophobic character of the RRIL-1 **2**, RRIL-2 **3** and RRLL **4** peptide promote NET formation through destabilization of the phagolysosomal membrane as described by Reithofer et al. and Neumann et al. [57,82]. Upon encapsulation of these peptides within silica, their positive charge is reduced and their hydrophobic residues are masked, thus decreasing their ability to trigger NET release. Due to their negative charge, calcinated silica particles fail to activate neutrophils to release NETs. The cationic CysR5 peptide **1** is unable to initiate NET release because it lacks the hydrophobic character that is needed to disrupt the lysosomal membrane. Furthermore, the neutral silica particles did not promote NET formation and we were able to confirm that particle size and shape do not influence the induction of NET as reported by Desai et al. [86]. Owing to the small sample size (*n* = 4) and high variance of our results due to highly individual immune responses, additional studies have to be performed to confirm this trend. However, we believe that our study can pave the way for the development of biomimetic, positively charged, biocompatible particles that offer a good safety profile as Bialas et al. have already demonstrated that R5-based silica nanoparticles were taken up in human colon adenocarcinoma HT-29 cells without any detectable cytotoxic effects at concentrations up to 100 µg/mL [67].

**Figure 9:**
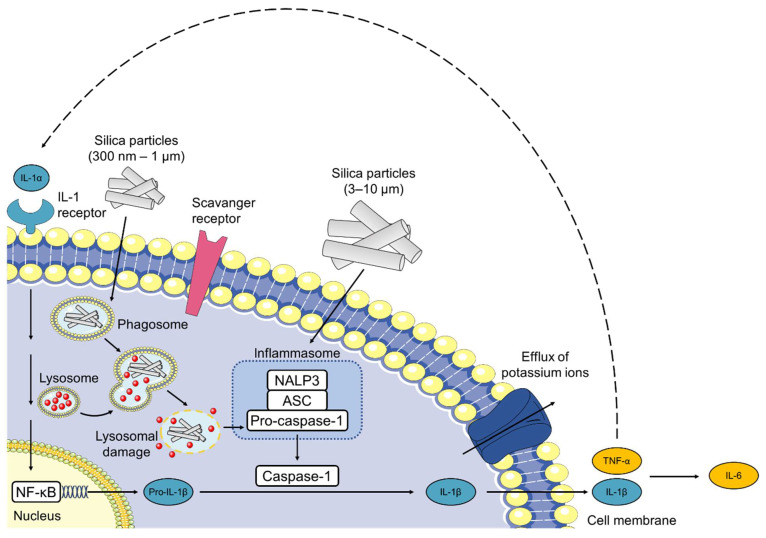
Silica particles activate the inflammasome and induce IL-1 production. Phagocytosis of silica particles leads to phagosomal damage and activation of NALP3. Then, NALP3 associates with the intracellular apoptosis-associated speck-like protein containing a caspase recruitment domain (ASC) that activates pro-caspase-1. The resulting inflammasome then cleaves pro-IL-1β to proinflammatory IL-1β. Binding of silica to the cell membrane can also induce secretion of IL-1β without lysosomal damage. Silica activates the NALP3 inflammasome that results in the efflux of potassium ions, suggesting an interaction of silica with a membrane-associated protein. Secretion of IL-1β and TNF-α leads to a positive feedback loop via IL-1 receptor signaling and additionally induces the production of IL-6. NALP3, NACHT, LRR, and PYD domains-containing protein 3; ASC, apoptosis-associated speck-like protein containing a caspase recruitment domain; NF-κB, nuclear factor-κB; IL, interleukin. This figure was created using Servier Medical Art templates, which are licensed under a Creative Commons Attribution 3.0 Unported License; https://smart.servier.com, accessed on 7 June 2022.

In our study, silica nanoparticles induced the specific production of the pro-inflammatory cytokines IL-6 and TNF-α in murine bone s, similar to previous studies [87,88,89,90,91,92,93]. In general, the secretion of pro-inflammatory cytokines is induced upon activation of pattern recognition receptors (PRRs) [94,95]. Silica crystals, calcium phosphate, alum and other particulates are internalized via phagocytosis and stimulate the NOD-like receptor and intracellular PRR, NLRP3 (previously termed NALP3) as shown in Figure 9 [95,96,97]. 

However, not only the direct sensing of the crystal structure, but also lysosomal perturbation seem to lead to NLRP3 activation [96]. In addition, the inflammatory responses do not appear to be limited to crystalline particles. Amorphous (non-crystalline) nano-, submicro- and micro-sized silica particles have also induced the secretion of IL-1β via activation of the inflammasome [98,99,100]. After internalization, silica particles have been reported to destabilize and disrupt the lysosomal membrane, thereby triggering the release of lysosomal content including, cathepsin B, into the cytosol and causing NLRP3 inflammasome activation [96]. Furthermore, the presence of cytoplasmic ROS may play an important role in the activation of the NLRP3 inflammasome as reported by several authors. Nevertheless, it is still unclear whether these phenomena occur independently or in a coordinated manner and how the endosomal rupture is facilitated [96,101]. Some studies suggest that the internalization of silica nanoparticles leads to the generation of ROS, thus activating the NLRP3 inflammasome and inducing IL-1β and TNF-α release [94,102,103,104]. Stimulation of BMDCs with cytokines such as IL-1β and TNF-α subsequently leads to the production of IL-6 and a positive feedback loop via IL-1 receptor signalling [105,106,107].

## 5. Conclusions

Here, we have shown that silaffin-derived R5 and RRIL peptides as well as biomimetic silica particles lead to the formation of NETs in human neutrophils and induce IL-6 and TNF-α secretion in murine bone marrow-derived dendritic cells. The R5 peptide and the corresponding RRIL motif are derived from the diatom *Cylindrotheca fusiformis* (*C. fusiformis*) silaffin protein [47,48,49] and can biomimetically precipitate silica particles of different morphologies from silicic acid at neutral pH and room temperature in aqueous solution without the need for surfactants or organic templates that have to be removed by calcination at high temperatures as it is necessary for the synthesis mesoporous silica nanoparticles (MSNs) [47,48,49,50,50,51,52]. Therefore, sensitive cargo such as peptide antigens, folded proteins or oligonucleotides can be directly conjugated to R5 and RRIL peptides and encapsulated into silica particles. These results show the potential of silaffin-derived peptides and silica particles as a self-adjuvanting platform for future (subunit) vaccines. However, an in-depth analysis of the underlying pathway of activation is needed to elucidate the mechanisms behind the immunomodulatory effects of silaffin-derived biomimetic silica particles. Furthermore, we provided the first biophysical characterization of the silaffin-derived biomimetic silica particles as well as insights into the release kinetics of silica encapsulated peptide–antigen conjugates. These results might pave the way for novel routes of administration by overcoming obstacles associated with oral delivery, such as antigen degradation in the harsh gastrointestinal environment with a wide range of pH, poor membrane permeability, and inefficient uptake by APCs. Thus, the drawbacks of subcutaneous or intramuscular injection including invasive procedures, poor patient compliance and the requirement of professional personnel could be circumvented, leading to an ease of administration, less stress and pain for the patients, minimal risk of contamination and cost benefits.

## Figures and Tables

**Table 1:** Sequences of silica-precipitating peptides and peptide incorporation efficiency. * Indicates branching via the lysine (K) sidechain.

Peptide	Compound	Sequence	Incorporation Efficiency/%
CysR5	**1**	H-CSSKKSGSYSGSKGSKRRIL-OH	84.4 ± 4.3
RRIL-1	**2**	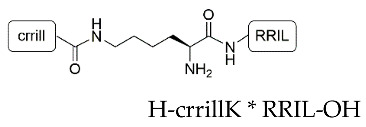	84.0 ± 5.4
RRIL-2	**3**	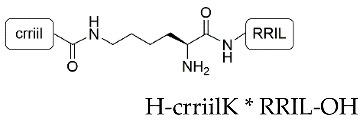	89.0 ± 0.6
RRLL	**4**	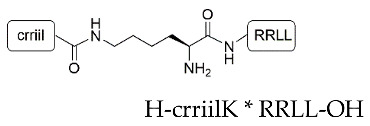	95.3 ± 2.8

**Table 2:** Peptide incorporation efficiency of peptide–antigen conjugates.

Peptide Conjugate	Compound	Incorporation Efficiency/%
CysR5-Bet v 1	**1b**	42.7 ± 7.9
CysR5-Phl p 5	**1c**	32.4 ± 10.7
RRIL-2-Bet v 1	**3b**	89.9 ± 1.6
RRIL-2-Phl p 5	**3c**	85.3 ± 1.6

**Table 3:** Physicochemical properties of the silica-based particles obtained from nitrogen adsorption (−196 °C) before and after calcination.

Sample	Compound	*S*_BET_^a^/m^2^ g^−1^	TPV ^b^/cm³ g^−1^	W_ads_^c^/nm
Before calcination				
CysR5	**1′**	13	0.01	n.d.
RRIL-1	**2′**	48	0.03	n.d.
RRIL-2	**3′**	22	0.01	n.d.
After calcination				
CysR5	**1** **″**	727	0.3	1.1
RRIL-1	**2** **″**	180	0.1	6.5
RRIL-2	**3** **″**	650	0.3	1.3

^a^ Specific surface area determined using the BET the Micropore BET Assistant was used. The points were chosen individually to ensure a negative c constant value and a linear BET regression. ^b^ Total pore volume calculated using the Gurvitch rule at P/P_0_ = 0.95. ^c^ Mode pore size calculated using the kernel of NLDFT metastable isotherms (adsorption branch).

## Data Availability

All data is available as supporting information to this article or upon request from the authors.

## Supplementary Materials

The following supporting information can be downloaded at: https://www.mdpi.com/article/10.3390/pharmaceutics15010121/s1, Figure S1: Final analysis of the CysR5 peptide; Figure S2. Final analysis of the RRIL-1 peptide; Figure S3. Final analysis of the RRIL-2 peptide; Figure S4. Final analysis of the RRLL peptide; Figure S5. EDX spectra of silica particles; Figure S6. Fluorescence images of human neutrophils; Figure S7. Final analysis of the P467 peptide; Figure S8. Final analysis of the Bet v 1 peptide; Figure S9. Final analysis of the Phl p 5 peptide; Figure S10. Final analysis of the CysR5-P467 peptide-antigen conjugate; Figure S11. Final analysis of the CysR5-Bet v 1 peptide-antigen conjugate; Figure S12. Final analysis of the CysR5-Phl p 5 peptide-antigen conjugate; Figure S13. Final analysis of the RRIL-1-P467 peptide-antigen conjugate; Figure S14. Final analysis of the RRIL-1-Bet v 1peptide-antigen conjugate; Figure S15. Final analysis of the RRIL-1-Phl p 5 peptide-antigen conjugate; Figure S16. Final analysis of the RRIL-2-P467 peptide-antigen conjugate; Figure S17. Final analysis of the RRIL-2-Bet v 1peptide-antigen conjugate; Figure S18. Final analysis of the RRIL-2-Phl p 5 peptide-antigen conjugate; Figure S19. Final analysis of the RRLL-P467 peptide-antigen conjugate; Figure S20. Final analysis of the RRLL-Bet v 1peptide-antigen conjugate; Figure S21. Final analysis of the RRLL-Phl p 5 peptide-antigen conjugate; Figure S22. Release of CysR5-Phl p 5 peptide-antigen conjugate 1c; Figure S23. Scanning electron micrographs (SEM) of silica particles; Figure S24. Zeta potential of CysR5 (1′), RRIL-1 (2′), RRIL-2 (3′) and RRLL (4′) silica particles; Figure S25. Pore size distribution; Table S1. Calculated via ProtParam by Expas.

## References

[1] Fenner F., Plotkin S.A. (2011). Smallpox Eradication: The Vindication of Jenner’s Prophesy. History of Vaccine Development.

[2] Plotkin S.A. (2009). Vaccines: The Fourth Century. Clin. Vaccine Immunol..

[3] Moyle P.M., Toth I. (2008). Self-Adjuvanting Lipopeptide Vaccines. Curr. Med. Chem..

[4] (2010). Possible Applications for Replicating HIV-1 Vectors. HIV Ther..

[5] Kaufmann S.H., Juliana McElrath M., Lewis D.J., Del Giudice G. (2014). Challenges and Responses in Human Vaccine Development. Curr. Opin. Immunol..

[6] Pulendran B., Ahmed R. (2011). Immunological Mechanisms of Vaccination. Nat. Immunol..

[7] Boland G., Beran J., Lievens M., Sasadeusz J., Dentico P., Nothdurft H., Zuckerman J.N., Genton B., Steffen R., Loutan L. (2004). Safety and Immunogenicity Profile of an Experimental Hepatitis B Vaccine Adjuvanted with AS04. Vaccine.

[8] Giannini S.L., Hanon E., Moris P., Van Mechelen M., Morel S., Dessy F., Fourneau M.A., Colau B., Suzich J., Losonksy G. (2006). Enhanced Humoral and Memory B Cellular Immunity Using HPV16/18 L1 VLP Vaccine Formulated with the MPL/Aluminium Salt Combination (AS04) Compared to Aluminium Salt Only. Vaccine.

[9] Vesikari T., Groth N., Karvonen A., Borkowski A., Pellegrini M. (2009). MF59®-Adjuvanted Influenza Vaccine (FLUAD^®^) in Children: Safety and Immunogenicity Following a Second Year Seasonal Vaccination. Vaccine.

[10] Ott G., Barchfeld G.L., Nest G.V. (1995). Enhancement of Humoral Response against Human Influenza Vaccine with the Simple Submicron Oil/Water Emulsion Adjuvant MF59. Vaccine.

[11] Durando P., Fenoglio D., Boschini A., Ansaldi F., Icardi G., Sticchi L., Renzoni A., Fabbri P., Ferrera A., Parodi A. (2008). Safety and Immunogenicity of Two Influenza Virus Subunit Vaccines, with or without MF59 Adjuvant, Administered to Human Immunodeficiency Virus Type 1-Seropositive and -Seronegative Adults. Clin. Vaccine Immunol..

[12] Chu D.W.-S., Hwang S.-J., Lim F.S., Oh H.M.L., Thongcharoen P., Yang P.-C., Bock H.L., Dramé M., Gillard P., Hutagalung Y. (2009). Immunogenicity and Tolerability of an AS03A-Adjuvanted Prepandemic Influenza Vaccine: A Phase III Study in a Large Population of Asian Adults. Vaccine.

[13] Schwarz T.F., Horacek T., Knuf M., Damman H.-G., Roman F., Dramé M., Gillard P., Jilg W. (2009). Single Dose Vaccination with AS03-Adjuvanted H5N1 Vaccines in a Randomized Trial Induces Strong and Broad Immune Responsiveness to Booster Vaccination in Adults. Vaccine.

[14] World Health Organization (1997). Biotechnology and World Health : Risks and Benefits of Vaccines and Other Medical Products Produced by Genetic Engineering : Proceedings of a WHO Meeting.

[15] Clements C.J., Griffiths E. (2002). The Global Impact of Vaccines Containing Aluminium Adjuvants. Vaccine.

[16] Glenny A.T., Pope C.G., Waddington H., Wallace U. (1926). Immunological Notes. XVII–XXIV. J. Pathol. Bacteriol..

[17] HogenEsch H., O’Hagan D.T., Fox C.B. (2018). Optimizing the Utilization of Aluminum Adjuvants in Vaccines: You Might Just Get What You Want. Npj Vaccines.

[18] Eisenbarth S.C., Colegio O.R., O’Connor W., Sutterwala F.S., Flavell R.A. (2008). Crucial Role for the Nalp3 Inflammasome in the Immunostimulatory Properties of Aluminium Adjuvants. Nature.

[19] Marrack P., McKee A.S., Munks M.W. (2009). Towards an Understanding of the Adjuvant Action of Aluminium. Nat. Rev. Immunol..

[20] Reed S.G., Bertholet S., Coler R.N., Friede M. (2009). New Horizons in Adjuvants for Vaccine Development. Trends Immunol..

[21] McKee A.S., MacLeod M.K., Kappler J.W., Marrack P. (2010). Immune Mechanisms of Protection: Can Adjuvants Rise to the Challenge?. BMC Biol..

[22] Aguilar J.C., Rodríguez E.G. (2007). Vaccine Adjuvants Revisited. Vaccine.

[23] Foged C., Hansen J., Agger E.M. (2012). License to Kill: Formulation Requirements for Optimal Priming of CD8+ CTL Responses with Particulate Vaccine Delivery Systems. Eur. J. Pharm. Sci..

[24] Nguyen T.L., Cha B.G., Choi Y., Im J., Kim J. (2020). Injectable Dual-Scale Mesoporous Silica Cancer Vaccine Enabling Efficient Delivery of Antigen/Adjuvant-Loaded Nanoparticles to Dendritic Cells Recruited in Local Macroporous Scaffold. Biomaterials.

[25] Min Y., Roche K.C., Tian S., Eblan M.J., McKinnon K.P., Caster J.M., Chai S., Herring L.E., Zhang L., Zhang T. (2017). Antigen-Capturing Nanoparticles Improve the Abscopal Effect and Cancer Immunotherapy. Nat. Nanotechnol..

[26] Kuai R., Ochyl L.J., Bahjat K.S., Schwendeman A., Moon J.J. (2017). Designer Vaccine Nanodiscs for Personalized Cancer Immunotherapy. Nat. Mater..

[27] Singh A., Peppas N.A. (2014). Hydrogels and Scaffolds for Immunomodulation. Adv. Mater..

[28] Treuel L., Jiang X., Nienhaus G.U. (2013). New Views on Cellular Uptake and Trafficking of Manufactured Nanoparticles. J. R. Soc. Interface.

[29] Wang D., Xu Z., Chen Z., Liu X., Hou C., Zhang X., Zhang H. (2014). Fabrication of Single-Hole Glutathione-Responsive Degradable Hollow Silica Nanoparticles for Drug Delivery. ACS Appl. Mater. Interfaces.

[30] Zhao S., Zhang S., Ma J., Fan L., Yin C., Lin G., Li Q. (2015). Double Loaded Self-Decomposable SiO_2_ Nanoparticles for Sustained Drug Release. Nanoscale.

[31] Zhang S., Chu Z., Yin C., Zhang C., Lin G., Li Q. (2013). Controllable Drug Release and Simultaneously Carrier Decomposition of SiO2-Drug Composite Nanoparticles. J. Am. Chem. Soc..

[32] Li A.W., Sobral M.C., Badrinath S., Choi Y., Graveline A., Stafford A.G., Weaver J.C., Dellacherie M.O., Shih T.-Y., Ali O.A. (2018). A Facile Approach to Enhance Antigen Response for Personalized Cancer Vaccination. Nat. Mater..

[33] Kim J., Li W.A., Choi Y., Lewin S.A., Verbeke C.S., Dranoff G., Mooney D.J. (2015). Injectable, Spontaneously Assembling, Inorganic Scaffolds Modulate Immune Cells in Vivo and Increase Vaccine Efficacy. Nat. Biotechnol..

[34] Dellacherie M.O., Li A.W., Lu B.Y., Mooney D.J. (2018). Covalent Conjugation of Peptide Antigen to Mesoporous Silica Rods to Enhance Cellular Responses. Bioconjug. Chem..

[35] Mody K.T., Popat A., Mahony D., Cavallaro A.S., Yu C., Mitter N. (2013). Mesoporous Silica Nanoparticles as Antigen Carriers and Adjuvants for Vaccine Delivery. Nanoscale.

[36] Li Z., Barnes J.C., Bosoy A., Stoddart J.F., Zink J.I. (2012). Mesoporous Silica Nanoparticles in Biomedical Applications. Chem. Soc. Rev..

[37] CFR-Code of Federal Regulations Title 21. https://www.accessdata.fda.gov/scripts/cdrh/cfdocs/cfCFR/CFRSearch.cfm?fr=172.480.

[38] Popplewell J.F., King S.J., Day J.P., Ackrill P., Fifield L.K., Cresswell R.G., di Tada M.L., Liu K. (1998). Kinetics of Uptake and Elimination of Silicic Acid by a Human Subject: A Novel Application of 32Si and Accelerator Mass Spectrometry. J. Inorg. Biochem..

[39] Croissant J.G., Fatieiev Y., Khashab N.M. (2017). Degradability and Clearance of Silicon, Organosilica, Silsesquioxane, Silica Mixed Oxide, and Mesoporous Silica Nanoparticles. Adv. Mater..

[40] Park J.-H., Gu L., von Maltzahn G., Ruoslahti E., Bhatia S.N., Sailor M.J. (2009). Biodegradable Luminescent Porous Silicon Nanoparticles for in Vivo Applications. Nat. Mater..

[41] Li X., Zhang L., Dong X., Liang J., Shi J. (2007). Preparation of Mesoporous Calcium Doped Silica Spheres with Narrow Size Dispersion and Their Drug Loading and Degradation Behavior. Microporous Mesoporous Mater..

[42] Rogalla S., Flisikowski K., Gorpas D., Mayer A.T., Flisikowska T., Mandella M.J., Ma X., Casey K.M., Felt S.A., Saur D. (2019). Biodegradable Fluorescent Nanoparticles for Endoscopic Detection of Colorectal Carcinogenesis. Adv. Funct. Mater..

[43] Choi Y., Lee J.E., Lee J.H., Jeong J.H., Kim J. (2015). A Biodegradation Study of SBA-15 Microparticles in Simulated Body Fluid and In Vivo. Langmuir.

[44] Strobl J., Kozak F., Kamalov M., Reichinger D., Kurzbach D., Becker C.F.W. (2022). Understanding Self-Assembly of Silica Precipitating Peptides to Control Silica. Adv. Mater..

[45] Calzetti F., Tamassia N., Arruda-Silva F., Gasperini S., Cassatella M.A. (2017). The Importance of Being “Pure” Neutrophils. J. Allergy Clin. Immunol..

[46] Drinić M., Wagner A., Sarate P., Zwicker C., Korb E., Loupal G., Peschke R., Joachim A., Wiedermann U., Schabussova I. (2017). Toxoplasma Gondii Tachyzoite-Extract Acts as a Potent Immunomodulator against Allergic Sensitization and Airway Inflammation. Sci. Rep..

[47] Kröger N., Deutzmann R., Sumper M. (1999). Polycationic Peptides from Diatom Biosilica That Direct Silica Nanosphere Formation. Science.

[48] Kröger N., Deutzmann R., Sumper M. (2001). Silica-Precipitating Peptides from Diatoms The Chemical Structure of Silaffin-1A from Cylindrotheca Fusiformis. J. Biol. Chem..

[49] Kamalov M., Capel P.D., Rentenberger C., Müllner A.R.M., Peterlik H., Becker C.F.W. (2018). Silaffin-Inspired Peptide Assemblies Template Silica Particles with Variable Morphologies. ChemNanoMat.

[50] Lechner C.C., Becker C.F.W. (2015). Silaffins in Silica Biomineralization and Biomimetic Silica Precipitation. Mar. Drugs.

[51] Lechner C.C., Becker C.F.W. (2013). Modified Silaffin R5 Peptides Enable Encapsulation and Release of Cargo Molecules from Biomimetic Silica Particles. Bioorg. Med. Chem..

[52] Lechner C.C., Becker C.F. (2015). Immobilising Proteins on Silica with Site-Specifically Attached Modified Silaffin Peptides. Biomater. Sci..

[53] Lechner C.C., Becker C.F.W. (2014). A Sequence-Function Analysis of the Silica Precipitating Silaffin R5 Peptide. J. Pept. Sci..

[54] Brinkmann V., Reichard U., Goosmann C., Fauler B., Uhlemann Y., Weiss D.S., Weinrauch Y., Zychlinsky A. (2004). Neutrophil Extracellular Traps Kill Bacteria. Science.

[55] Stephen J., Scales H.E., Benson R.A., Erben D., Garside P., Brewer J.M. (2017). Neutrophil Swarming and Extracellular Trap Formation Play a Significant Role in Alum Adjuvant Activity. Npj Vaccines.

[56] Munks M.W., McKee A.S., MacLeod M.K., Powell R.L., Degen J.L., Reisdorph N.A., Kappler J.W., Marrack P. (2010). Aluminum Adjuvants Elicit Fibrin-Dependent Extracellular Traps In Vivo. Blood.

[57] Reithofer M., Karacs J., Strobl J., Kitzmüller C., Polak D., Seif K., Kamalov M., Becker C.F.W., Greiner G., Schmetterer K. (2020). Alum Triggers Infiltration of Human Neutrophils Ex Vivo and Causes Lysosomal Destabilization and Mitochondrial Membrane Potential-Dependent NET-Formation. FASEB J..

[58] Douda D.N., Khan M.A., Grasemann H., Palaniyar N. (2015). SK3 Channel and Mitochondrial ROS Mediate NADPH Oxidase-Independent NETosis Induced by Calcium Influx. Proc. Natl. Acad. Sci. USA.

[59] Gajhede M., Osmark P., Poulsen F.M., Ipsen H., Larsen J.N., van Neerven R.J.J., Schou C., Løwenstein H., Spangfort M.D. (1996). X-ray and NMR Structure of Bet v 1, the Origin of Birch Pollen Allergy. Nat. Struct. Biol..

[60] Ebner C., Hirschwehr R., Bauer L., Breiteneder H., Valenta R., Ebner H., Kraft D., Scheiner O. (1995). Identification of Allergens in Fruits and Vegetables: IgE Cross-Reactivities with the Important Birch Pollen Allergens Bet v 1 and Bet v 2 (Birch Profilin). J. Allergy Clin. Immunol..

[61] Hufnagl K., Winkler B., Focke M., Valenta R., Scheiner O., Renz H., Wiedermann U. (2005). Intranasal Tolerance Induction with Polypeptides Derived from 3 Noncross-Reactive Major Aeroallergens Prevents Allergic Polysensitization in Mice. J. Allergy Clin. Immunol..

[62] Vrtala S., Sperr W.R., Reimitzer I., van Ree R., Laffer S., Müller W.D., Valent P., Lechner K., Rumpold H., Kraft D. (1993). CDNA Cloning of a Major Allergen from Timothy Grass (Phleum Pratense) Pollen; Characterization of the Recombinant Phl PV Allergen. J. Immunol..

[63] Wagner S., Jasinska J., Breiteneder H., Kundi M., Pehamberger H., Scheiner O., Zielinski C.C., Wiedermann U. (2007). Delayed Tumor Onset and Reduced Tumor Growth Progression after Immunization with a Her-2/Neu Multi-Peptide Vaccine and IL-12 in c-Neu Transgenic Mice. Breast Cancer Res. Treat..

[64] Wiedermann U., Davis A.B., Zielinski C.C. (2013). Vaccination for the Prevention and Treatment of Breast Cancer with Special Focus on Her-2/Neu Peptide Vaccines. Breast Cancer Res. Treat..

[65] Tobias J., Garner-Spitzer E., Drinić M., Wiedermann U. (2022). Vaccination against Her-2/Neu, with Focus on Peptide-Based Vaccines. ESMO Open.

[66] Sulpizi M., Gaigeot M.-P., Sprik M. (2012). The Silica–Water Interface: How the Silanols Determine the Surface Acidity and Modulate the Water Properties. J. Chem. Theory Comput..

[67] Favero G.D., Bialas F., Grabher S., Wittig A., Bräuer B., Gerthsen D., Echalier C., Kamalov M., Marko D., Becker C.F.W. (2019). Silica Particles with a Quercetin–R5 Peptide Conjugate Are Taken up into HT-29 Cells and Translocate into the Nucleus. Chem. Commun..

[68] Sikora A., Bartczak D., Geißler D., Kestens V., Roebben G., Ramaye Y., Varga Z., Palmai M., Shard A.G., Goenaga-Infante H. (2015). A Systematic Comparison of Different Techniques to Determine the Zeta Potential of Silica Nanoparticles in Biological Medium. Anal. Methods.

[69] Al-Garawi Z.S., Kostakis G.E., Serpell L.C. (2016). Chemically and Thermally Stable Silica Nanowires with a β-Sheet Peptide Core for Bionanotechnology. J. Nanobiotechnology.

[70] von Baeckmann C., Guillet-Nicolas R., Renfer D., Kählig H., Kleitz F. (2018). A Toolbox for the Synthesis of Multifunctionalized Mesoporous Silica Nanoparticles for Biomedical Applications. ACS Omega.

[71] Möller K., Bein T. (2017). Talented Mesoporous Silica Nanoparticles. Chem. Mater..

[72] Thommes M., Kaneko K., Neimark A.V., Olivier J.P., Rodriguez-Reinoso F., Rouquerol J., Sing K.S.W. (2015). Physisorption of Gases, with Special Reference to the Evaluation of Surface Area and Pore Size Distribution (IUPAC Technical Report). Pure Appl. Chem..

[73] Cychosz K.A., Guillet-Nicolas R., García-Martínez J., Thommes M. (2017). Recent Advances in the Textural Characterization of Hierarchically Structured Nanoporous Materials. Chem. Soc. Rev..

[74] Schlumberger C., Thommes M. (2021). Characterization of Hierarchically Ordered Porous Materials by Physisorption and Mercury Porosimetry—A Tutorial Review. Adv. Mater. Interfaces.

[75] Rasmussen C.J., Vishnyakov A., Thommes M., Smarsly B.M., Kleitz F., Neimark A.V. (2010). Cavitation in Metastable Liquid Nitrogen Confined to Nanoscale Pores. Langmuir.

[76] Thommes M. (2010). Physical Adsorption Characterization of Nanoporous Materials. Chem. Ing. Tech..

[77] Landers J., Gor G.Y., Neimark A.V. (2013). Density Functional Theory Methods for Characterization of Porous Materials. Colloids Surf. Physicochem. Eng. Asp..

[78] Thommes M., Cychosz K.A. (2014). Physical Adsorption Characterization of Nanoporous Materials: Progress and Challenges. Adsorption.

[79] Juère E., Kleitz F. (2018). On the Nanopore Confinement of Therapeutic Drugs into Mesoporous Silica Materials and Its Implications. Microporous Mesoporous Mater..

[80] Velazquez-Salinas L., Verdugo-Rodriguez A., Rodriguez L.L., Borca M.V. (2019). The Role of Interleukin 6 During Viral Infections. Front. Microbiol..

[81] Angelone D.F., Wessels M.R., Coughlin M., Suter E.E., Valentini P., Kalish L.A., Levy O. (2006). Innate Immunity of the Human Newborn Is Polarized Toward a High Ratio of IL-6/TNF-α Production In Vitro and In Vivo. Pediatr. Res..

[82] Neumann A., Berends E.T.M., Nerlich A., Molhoek E.M., Gallo R.L., Meerloo T., Nizet V., Naim H.Y., von Köckritz-Blickwede M. (2014). The Antimicrobial Peptide LL-37 Facilitates the Formation of Neutrophil Extracellular Traps. Biochem. J..

[83] Hosoda H., Nakamura K., Hu Z., Tamura H., Reich J., Kuwahara-Arai K., Iba T., Tabe Y., Nagaoaka I. (2017). Antimicrobial Cathelicidin Peptide LL-37 Induces NET Formation and Suppresses the Inflammatory Response in a Mouse Septic Model. Mol. Med. Rep..

[84] Gorgojo J., Scharrig E., Gómez R.M., Harvill E.T., Rodríguez M.E. (2017). Bordetella Parapertussis Circumvents Neutrophil Extracellular Bactericidal Mechanisms. PLoS ONE.

[85] Oren Z., Lerman J.C., Gudmundsson G.H., Agerberth B., Shai Y. (1999). Structure and Organization of the Human Antimicrobial Peptide LL-37 in Phospholipid Membranes: Relevance to the Molecular Basis for Its Non-Cell-Selective Activity. Biochem. J..

[86] Desai J., Foresto-Neto O., Honarpisheh M., Steiger S., Nakazawa D., Popper B., Buhl E.M., Boor P., Mulay S.R., Anders H.-J. (2017). Particles of Different Sizes and Shapes Induce Neutrophil Necroptosis Followed by the Release of Neutrophil Extracellular Trap-like Chromatin. Sci. Rep..

[87] Kang K., Lim J.-S. (2012). Induction of Functional Changes of Dendritic Cells by Silica Nanoparticles. Immune Netw..

[88] Vallhov H., Gabrielsson S., Strømme M., Scheynius A., Garcia-Bennett A.E. (2007). Mesoporous Silica Particles Induce Size Dependent Effects on Human Dendritic Cells. Nano Lett..

[89] Mamaeva V., Sahlgren C., Lindén M. (2013). Mesoporous Silica Nanoparticles in Medicine—Recent Advances. Adv. Drug Deliv. Rev..

[90] Heidegger S., Gößl D., Schmidt A., Niedermayer S., Argyo C., Endres S., Bein T., Bourquin C. (2015). Immune Response to Functionalized Mesoporous Silica Nanoparticles for Targeted Drug Delivery. Nanoscale.

[91] Meraz I.M., Melendez B., Gu J., Wong S.T.C., Liu X., Andersson H.A., Serda R.E. (2012). Activation of the Inflammasome and Enhanced Migration of Microparticle-Stimulated Dendritic Cells to the Draining Lymph Node. Mol. Pharm..

[92] Skuland T., Låg M., Gutleb A.C., Brinchmann B.C., Serchi T., Øvrevik J., Holme J.A., Refsnes M. (2020). Pro-Inflammatory Effects of Crystalline- and Nano-Sized Non-Crystalline Silica Particles in a 3D Alveolar Model. Part. Fibre Toxicol..

[93] Napierska D., Thomassen L.C.J., Vanaudenaerde B., Luyts K., Lison D., Martens J.A., Nemery B., Hoet P.H.M. (2012). Cytokine Production by Co-Cultures Exposed to Monodisperse Amorphous Silica Nanoparticles: The Role of Size and Surface Area. Toxicol. Lett..

[94] Pétrilli V., Dostert C., Muruve D.A., Tschopp J. (2007). The Inflammasome: A Danger Sensing Complex Triggering Innate Immunity. Curr. Opin. Immunol..

[95] (2009). Innate Instruction of Adaptive Immunity Revisited: The Inflammasome. EMBO Mol. Med..

[96] Hornung V., Bauernfeind F., Halle A., Samstad E.O., Kono H., Rock K.L., Fitzgerald K.A., Latz E. (2008). Silica Crystals and Aluminum Salts Activate the NALP3 Inflammasome through Phagosomal Destabilization. Nat. Immunol..

[97] Sharp F.A., Ruane D., Claass B., Creagh E., Harris J., Malyala P., Singh M., O’Hagan D.T., Pétrilli V., Tschopp J. (2009). Uptake of Particulate Vaccine Adjuvants by Dendritic Cells Activates the NALP3 Inflammasome. Proc. Natl. Acad. Sci. USA.

[98] Sandberg W.J., Låg M., Holme J.A., Friede B., Gualtieri M., Kruszewski M., Schwarze P.E., Skuland T., Refsnes M. (2012). Comparison of Non-Crystalline Silica Nanoparticles in IL-1β Release from Macrophages. Part. Fibre Toxicol..

[99] Winter M., Beer H.-D., Hornung V., Krämer U., Schins R.P.F., Förster I. (2011). Activation of the Inflammasome by Amorphous Silica and TiO2 Nanoparticles in Murine Dendritic Cells. Nanotoxicology.

[100] Kojima S., Negishi Y., Tsukimoto M., Takenouchi T., Kitani H., Takeda K. (2014). Purinergic Signaling via P2X7 Receptor Mediates IL-1β Production in Kupffer Cells Exposed to Silica Nanoparticle. Toxicology.

[101] Kusaka T., Nakayama M., Nakamura K., Ishimiya M., Furusawa E., Ogasawara K. (2014). Effect of Silica Particle Size on Macrophage Inflammatory Responses. PLoS ONE.

[102] Yazdi A.S., Guarda G., Riteau N., Drexler S.K., Tardivel A., Couillin I., Tschopp J. (2010). Nanoparticles Activate the NLR Pyrin Domain Containing 3 (Nlrp3) Inflammasome and Cause Pulmonary Inflammation through Release of IL-1α and IL-1β. Proc. Natl. Acad. Sci. USA.

[103] Fubini B., Hubbard A. (2003). Reactive Oxygen Species (ROS) and Reactive Nitrogen Species (RNS) Generation by Silica in Inflammation and Fibrosis. Free Radic. Biol. Med..

[104] Joshi G.N., Knecht D.A. (2013). Silica Phagocytosis Causes Apoptosis and Necrosis by Different Temporal and Molecular Pathways in Alveolar Macrophages. Apoptosis.

[105] Xu Y.-D., Cheng M., Shang P.-P., Yang Y.-Q. (2022). Role of IL-6 in Dendritic Cell Functions. J. Leukoc. Biol..

[106] Herseth J.I., Refsnes M., Låg M., Schwarze P.E. (2009). Role of IL-1β and COX2 in Silica-Induced IL-6 Release and Loss of Pneumocytes in Co-Cultures. Toxicol. In Vitro.

[107] Franchi L., Eigenbrod T., Núñez G. (2009). Cutting Edge: TNF-α Mediates Sensitization to ATP and Silica via the NLRP3 Inflammasome in the Absence of Microbial Stimulation. J. Immunol..

