# Supplementary material for: A Biomimetic, Silaffin R5-Based Antigen Delivery Platform
Source: Pharmaceutics. 2022 Dec 29;15(1):121. doi: 10.3390/pharmaceutics15010121 (PMC9866965; doi:10.3390/pharmaceutics15010121)
Supplement: Supplementary file 1 [file pharmaceutics-15-00121-s001.zip › pharmaceutics-2095367-supplementary.pdf]

A biomimetic, silaffin R5-based antigen delivery platform

Supplementary information

Analytical data of synthetic peptides

The CysR5 peptide **1** was synthesized successfully as described and obtained in 61% yield from crude. The analytical data is shown in Fig.S1. The mass spectrum shows the three peaks for the $[M+4H]^{4+}$ (m/z (calc.): 530.11), $[M+3H]^{3+}$ (m/z (calc.): 706.48) and $[M+2H]^{2+}$ (m/z (calc.): 1059.22). The HPLC chromatogram shows a single peak with a retention time of 14.5 min indicting that the peptide was obtained in high (>95%) purity.

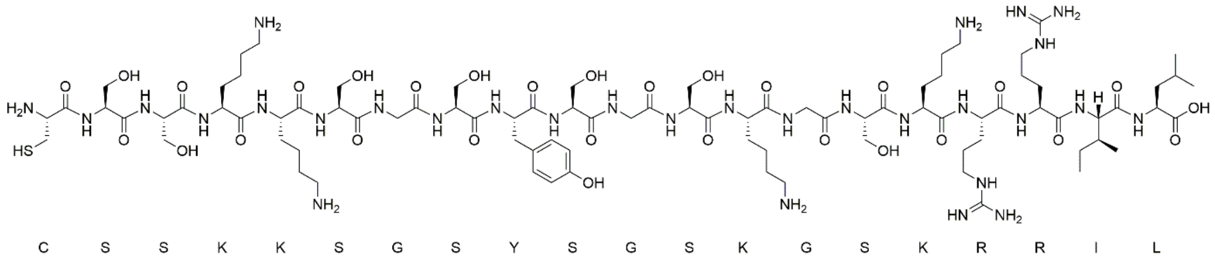

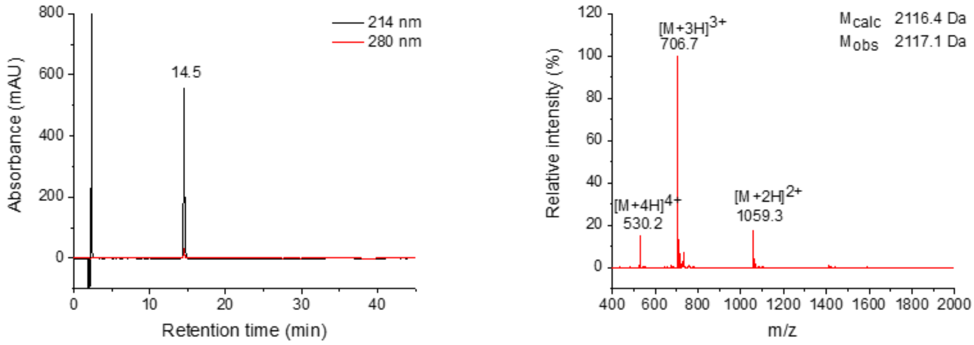

Figure S1. Final analysis of the CysR5 peptide. The analytical HPLC chromatogram (left) and the ESI-MS spectrum (right) of the purified peptide.

The RRIL-1 peptide **2** was synthesized successfully as described and obtained in 67% yield from crude. The analytical data is shown in Fig.S2. The mass spectrum shows the two peaks for the $[M+3H]^{3+}$ (m/z (calc.): 480.96) and$[M+2H]^{2+}$ (m/z (calc.): 720.94). The HPLC chromatogram shows a single peak with a retention time of 17.8 min indicting that the peptide was obtained in high (>95%) purity.

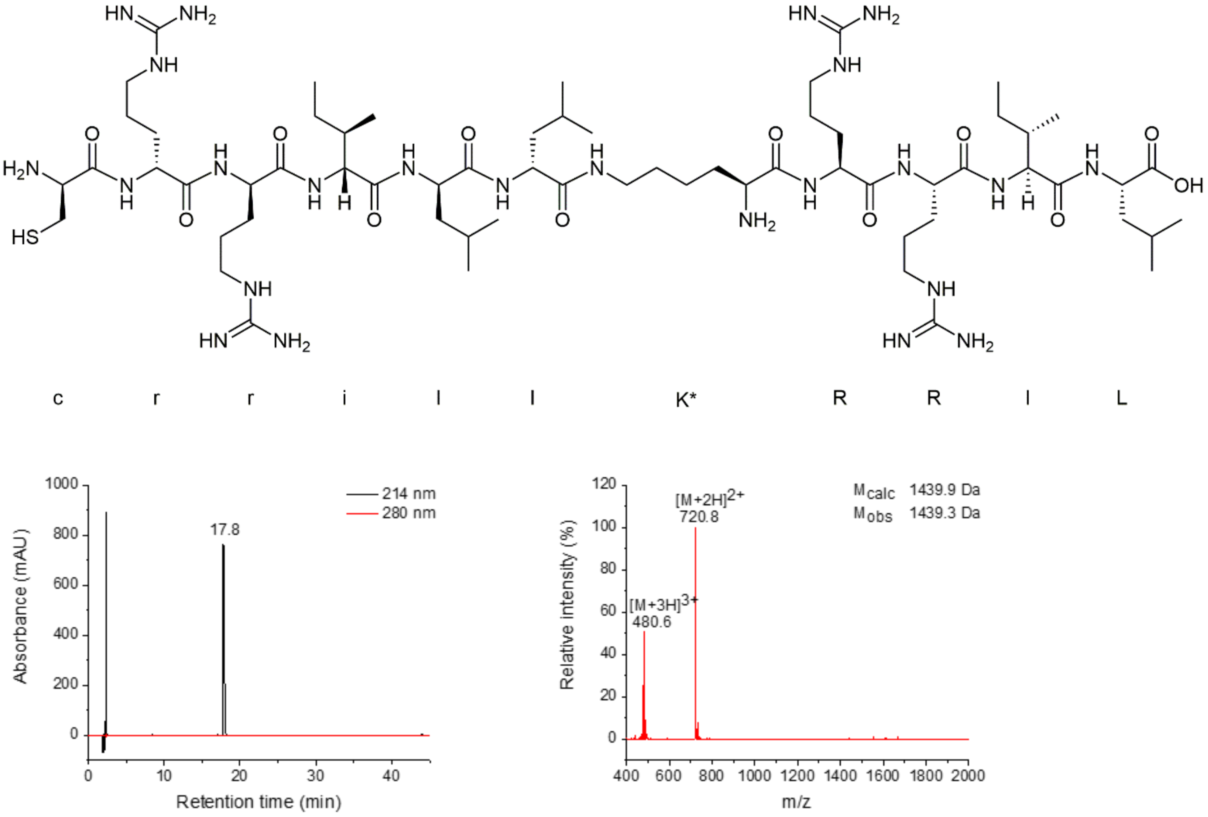

FigureS2. Final analysis of the RRIL-1 peptide. The analytical HPLC chromatogram (left) and the ESI-MS spectrum (right) of the purified peptide.

The RRIL-2 peptide **3** was synthesized successfully as described and obtained in 62% yield from crude. The analytical data is shown in Fig.S3. The mass spectrum shows the two peaks for the $[M+3H]^{3+}$ (m/z (calc.): 480.96) and$[M+2H]^{2+}$ (m/z (calc.): 720.94). The HPLC chromatogram shows a single peak with a retention time of 17.7 min indicting that the peptide was obtained in high (>95%) purity.

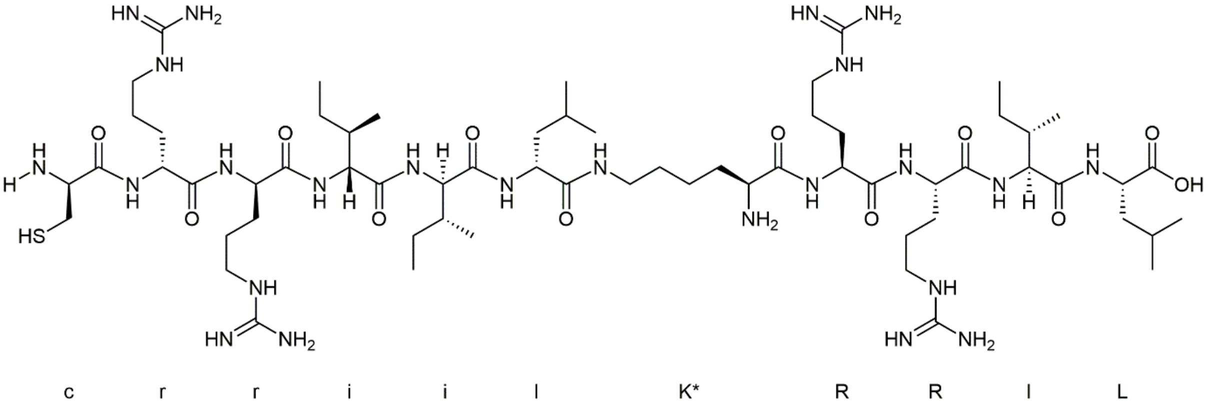

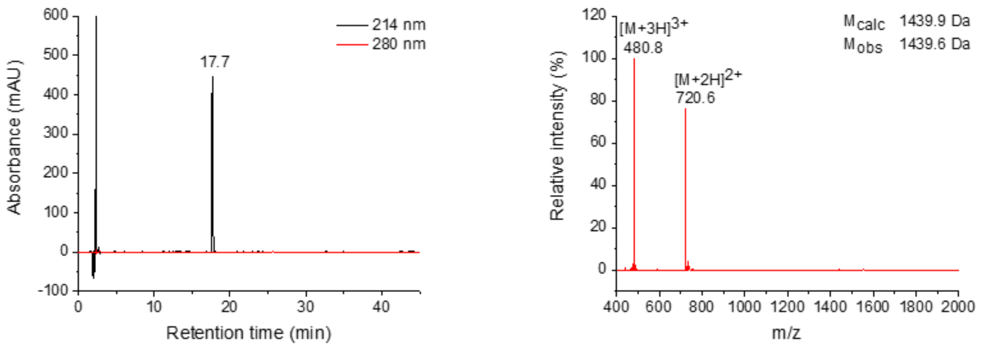

FigureS3. Final analysis of the RRIL-2 peptide. The analytical HPLC chromatogram (left) and the ESI-MS spectrum (right) of the purified peptide.

The RRLL peptide **4** was synthesized successfully as described and obtained in 72% yield from crude. The analytical data is shown in Fig.S4. The mass spectrum shows the two peaks for the $[M+3H]^{3+}$ (m/z (calc.): 480.96) and$[M+2H]^{2+}$ (m/z (calc.): 720.94). The HPLC chromatogram shows a single peak with a retention time of 16.5 min indicting that the peptide was obtained in high (>95%) purity.

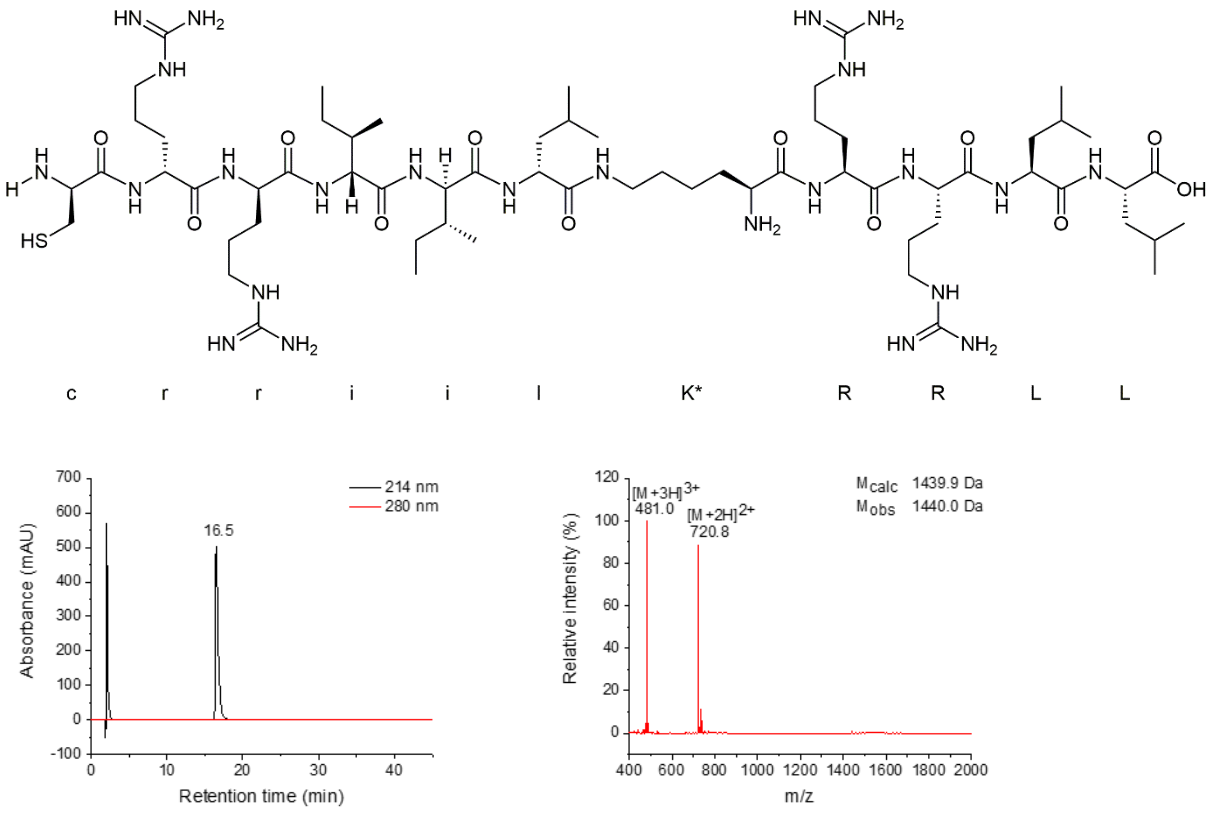

FigureS4. Final analysis of the RRLL peptide. The analytical HPLC chromatogram (left) and the ESI-MS spectrum (right) of the purified peptide.

Peptide incorporation efficiency

Peptides were incubated in 50 mM potassium phosphate buffer at pH 7 described above. After 24 h, 25 µl sample solution were mixed with 25 µl guanidinium chloride buffer (6 M GuHCl, 50 mM Tris-HCl pH 7.5) and injected into the HPLC system. Guanidinium chloride buffer was used to dissolve the precipitate to obtain comparable amounts of peptide. Silica precipitation was carried out as described above. After precipitation, 25 µl supernatant were mixed with 25 µl guanidinium chloride buffer and injected into the HPLC system. The area under the peak was calculated pre- and post-precipitation and a correction factor of 1.11 was used to compensate for the different amount of peptide injected after precipitation.

Energy dispersive X-ray spectroscopy

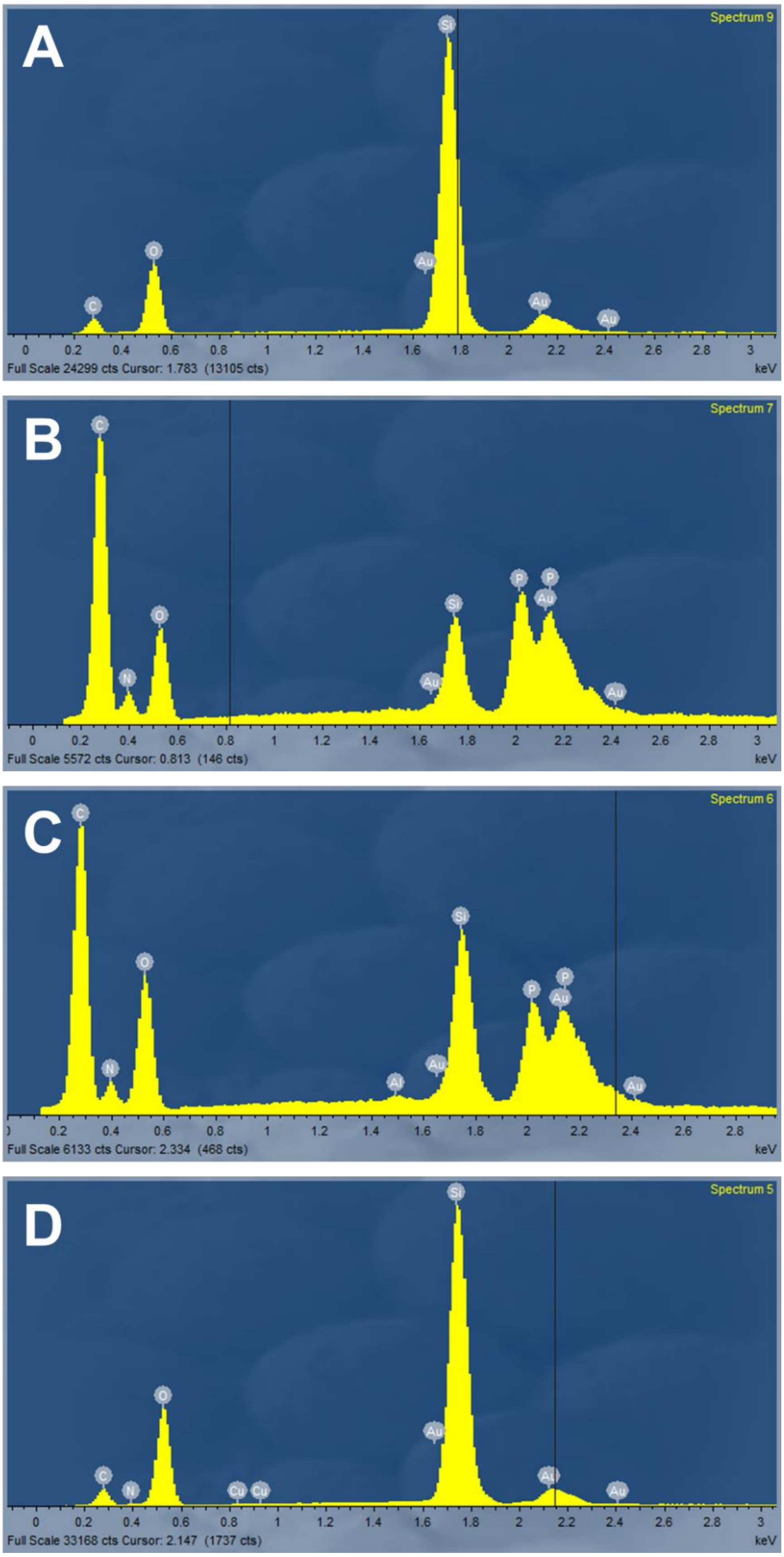

FigureS5. EDX spectra of silica particles generated from CysR5 **1'** (A), RRIL-1 **2'** (B), RRIL-2 **3'** (C) and RRLL **4'** (D). The element specific X-ray emission peak positions are indicated as grey circles. The vertical black line represents to cursor position during image acquisition.

Fluorescence microscopy

Neutrophils were seeded at a density of 1.5 × 10^6/mL in 96-well plates (Costar, Sigma-Aldrich) and primed with 25 ng/mL GM-CSF (PeproTech, Rocky Hill, NJ) in RPMI1640 with 2% autologous plasma for 30 minutes at 37°C and 5% CO2. Then, cells were stimulated with the positive control 5 µM ionomycin (Sigma-Aldrich) or the different stimuli at indicated concentrations for up to 3 hours. Medium alone served as negative control. Cells were fixed in 4% paraformaldehyde in PBS and permeabilized in PBS containing 5 mM NH_4Cl and 0.2% saponin (Sigma-Aldrich). Washing was performed three times with PBS for 5 minutes and blocking with 20% AB-serum in PBS for 60 minutes. Rabbit polyclonal antibody to Histone H3 (citrulline R2 + R8 + R17) (Abcam, Cambridge, UK) was added oN at 4°C and, as a secondary antibody, anti-mouse Alexa 568 (both Jackson Immuno Research Inc, West Grove, PA, USA) was added for 1 hour. DNA was visualized by incubation of the coverslips at RT with DRAQ5 (Invitrogen, Thermo Fisher Scientific) at 5 µM for 15 minutes. Fluorescence microscopy was performed with an Axioplan 2 microscope (Zeiss).

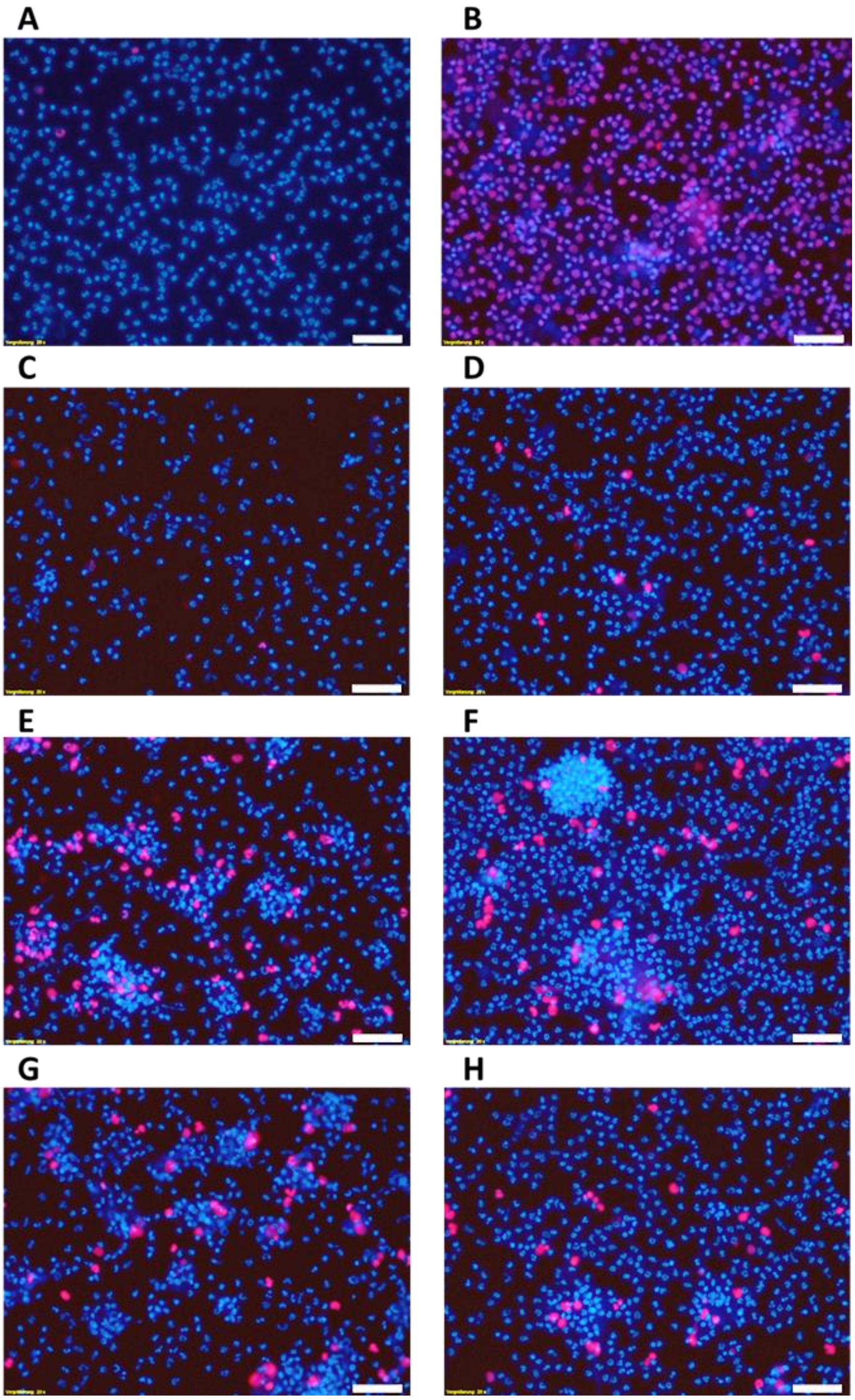

Figure S6. Fluorescence images of human neutrophils treated with (A) medium as negative control, (B) ionomycin as positive control, (C) RRIL-2 peptide **3**, (D) CysR5 peptide **1**, (E) RRIL-2 silica particles **3'**, (F) CysR5 silica particles **1'**, (G) calcinated RRIL-2 silica particles **3''** and (H) calcinated CysR5 silica particles **1''**. DNA was stained with DRAQ5 (blue) and citrullinated histone H3 was stained with Alexa Fluor 594 (red). Scale bars represent 50 µm.

Analytical data of synthetic peptide antigens

The P467 peptide **a** was synthesized successfully as described and obtained in 18% yield from crude. The analytical data is shown in Fig.S7. The mass spectrum shows the five peaks for the $[M+7H]^{7+}$ (m/z (calc.): 786.44), $[M+6H]^{6+}$ (m/z (calc.): 917.35), $[M+5H]^{5+}$ (m/z (calc.): 1100.62), $[M+4H]^{4+}$ (m/z (calc.): 1375.53) and $[M+3H]^{3+}$ (m/z (calc.): 1833.7). The HPLC chromatogram shows a single peak with a retention time of 22.2 min indicting that the peptide was obtained in high (>95%) purity.

H—P-E-S-F-D-G-D-P-A-S—N-T-A-P-L-Q-P-R-V-L—Q-G-L-P-R-E-Y-V-N-A—R-H-S-L-P-Y-M-P-I-W—K-F-P-D-E-E-G-A-C—OH

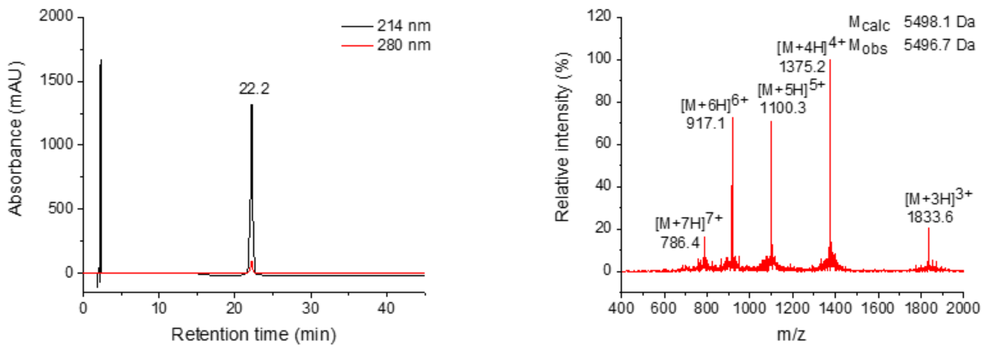

Figure S7 | Final analysis of the P467 peptide. The analytical HPLC chromatogram (left) and the ESI-MS spectrum (right) of the purified peptide.

The Bet v 1 peptide **b** was synthesized successfully as described and obtained in 27% yield from theory. The analytical data is shown in Fig.S8. The mass spectrum shows the two peaks for the $[M+2H]^{2+}$ (m/z (calc.): 822.44) and$[M+1H]^{+}$ (m/z (calc.): 1643.87). The HPLC chromatogram shows a single peak with a retention time of 22.0 min indicting that the peptide was obtained in high (>95%) purity.

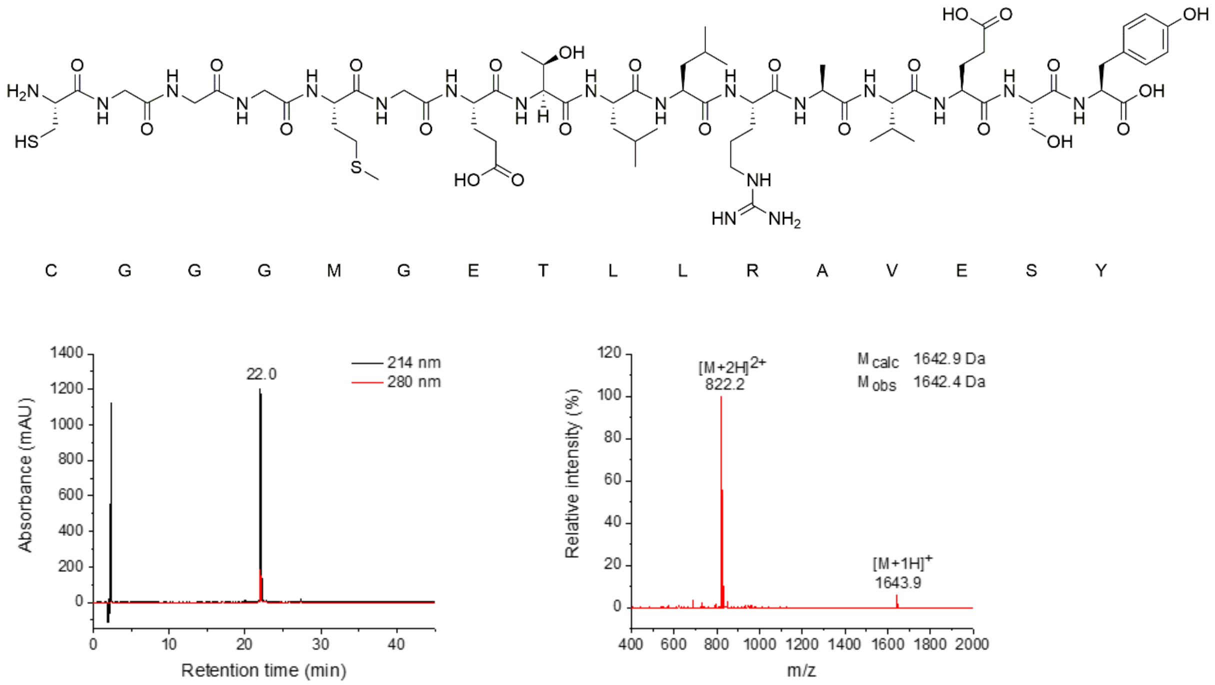

Figure S8 | Final analysis of the Bet v 1 peptide. The analytical HPLC chromatogram (left) and the ESI-MS spectrum (right) of the purified peptide.

The Phl p 5 peptide **c** was synthesized successfully as described and obtained in 26% yield from theory. The analytical data is shown in Fig.S9. The mass spectrum shows the two peaks for the $[M+3H]^{3+}$ (m/z (calc.): 748.53) and$[M+2H]^{2+}$ (m/z (calc.): 1122.29). The HPLC chromatogram shows a single peak with a retention time of 19.4 min indicting that the peptide was obtained in high (>95%) purity.

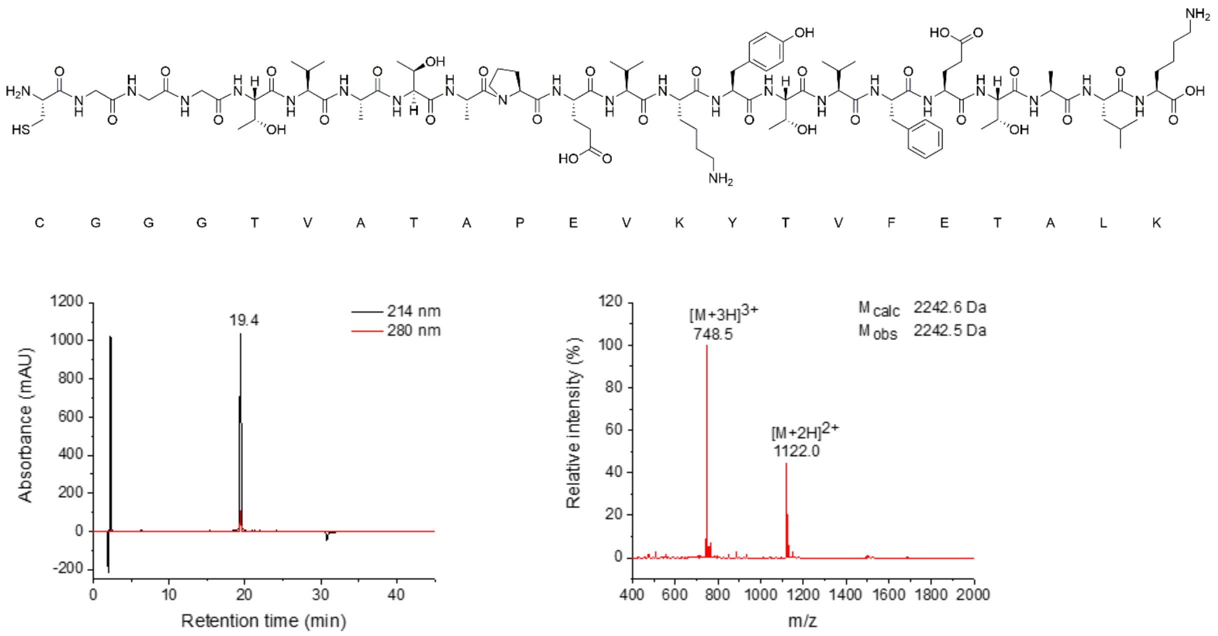

Figure S9 | Final analysis of the Phl p 5 peptide. The analytical HPLC chromatogram (left) and the ESI-MS spectrum (right) of the purified peptide.

Analytical data of synthetic peptide-antigen conjugates

The CysR5-P467 conjugate **1a** was synthesized successfully as described and obtained in 24% yield from theory. The analytical data is shown in Fig.S10. The mass spectrum shows the eight peaks for the $[M+11H]^{11+}$ (m/z (calc.): 693.05), $[M+10H]^{10+}$ (m/z (calc.): 762.26), $[M+9H]^{9+}$ (m/z (calc.): 846.84), $[M+8H]^{8+}$ (m/z (calc.): 952.57), $[M+7H]^{7+}$ (m/z (calc.): 1088.51), $[M+6H]^{6+}$ (m/z (calc.): 1269.77), $[M+5H]^{5+}$ (m/z (calc.): 1523.52) and $[M+4H]^{4+}$ (m/z (calc.): 1904.15). The HPLC chromatogram shows a single peak with a retention time of 22.2 min indicting that the peptide was obtained in high (>95%) purity.

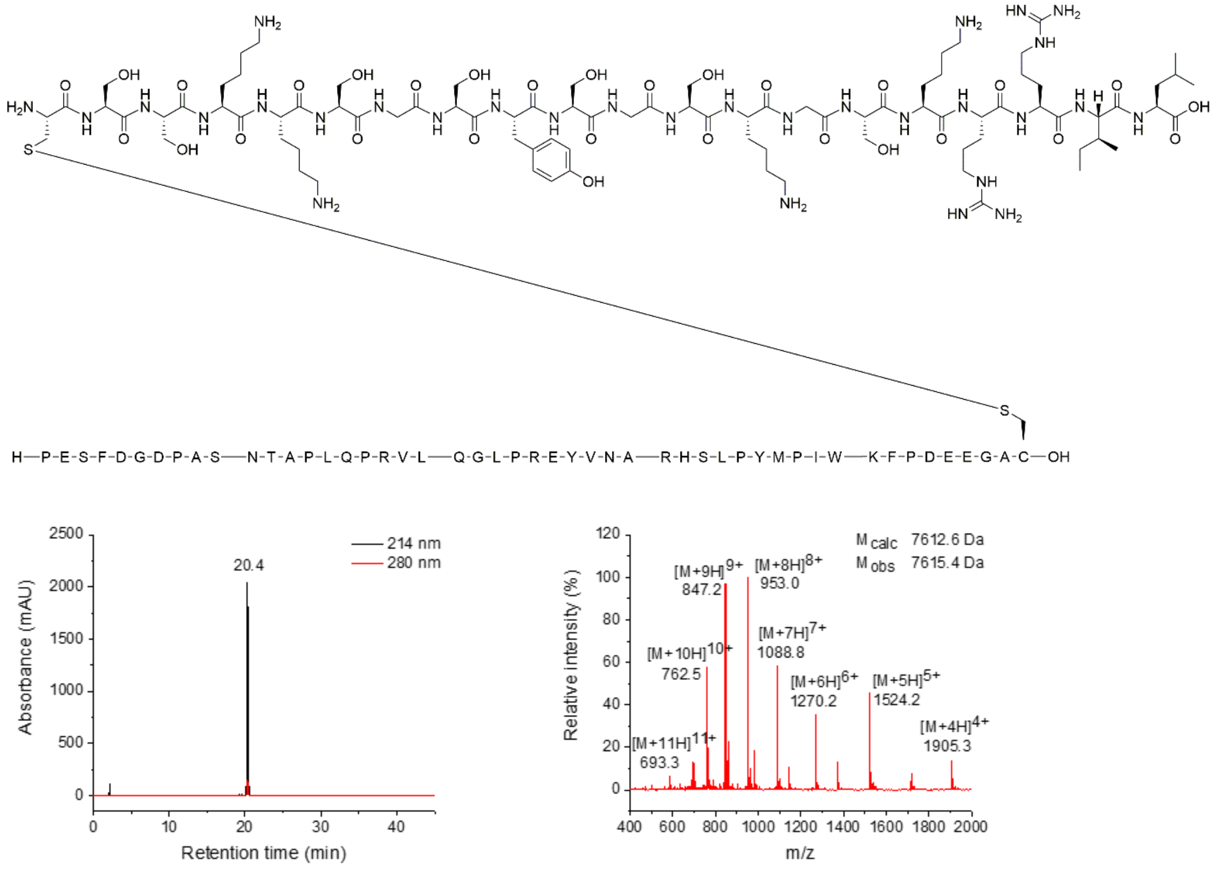

Figure S10 | Final analysis of the CysR5-P467 peptide-antigen conjugate. The analytical HPLC chromatogram (left) and the ESI-MS spectrum (right) of the purified conjugate.

The CysR5-Bet v 1 conjugate **1b** was synthesized successfully as described and obtained in 39% yield from theory. The analytical data is shown in Fig.S11. The mass spectrum shows the four peaks for the $[M+5H]^{5+}$ (m/z (calc.): 752.46), $[M+4H]^{4+}$ (m/z (calc.): 940.33), $[M+3H]^{3+}$ (m/z (calc.):1253.43) and $[M+2H]^{2+}$ (m/z (calc.):1879.65). The HPLC chromatogram shows a single peak with a retention time of 22.2 min indicting that the peptide was obtained in high (>95%) purity.

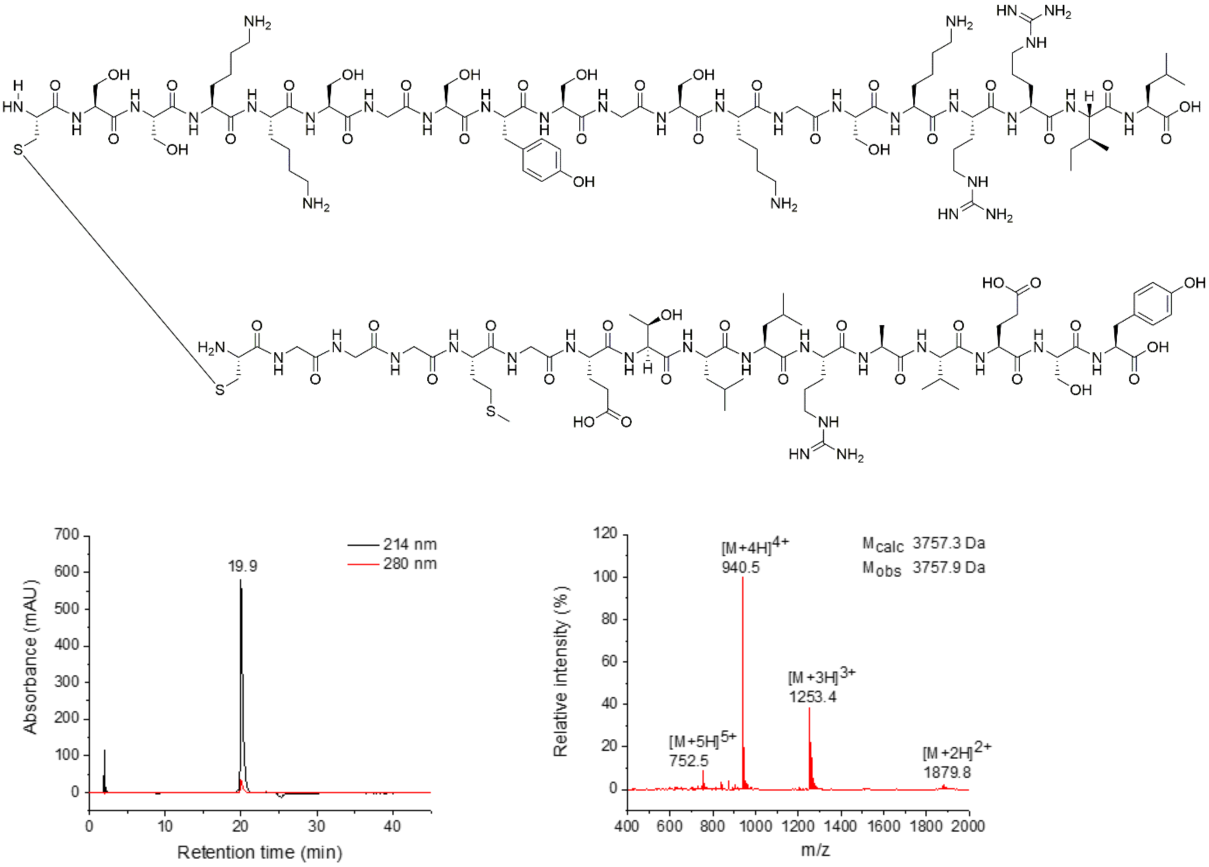

Figure S11 | Final analysis of the CysR5-Bet v 1 peptide-antigen conjugate. The analytical HPLC chromatogram (left) and the ESI-MS spectrum (right) of the purified conjugate.

The CysR5-Phl p 5 conjugate **1c** was synthesized successfully as described and obtained in 58% yield from theory. The analytical data is shown in Fig.S12. The mass spectrum shows the four peaks for the $[M+6H]^{6+}$ (m/z (calc.): 727.17), $[M+5H]^{5+}$ (m/z (calc.): 872.40), $[M+4H]^{4+}$ (m/z (calc.): 1090.25) and $[M+3H]^{3+}$ (m/z (calc.): 1453.33). The HPLC chromatogram shows a single peak with a retention time of 22.2 min indicting that the peptide was obtained in high (>95%) purity.

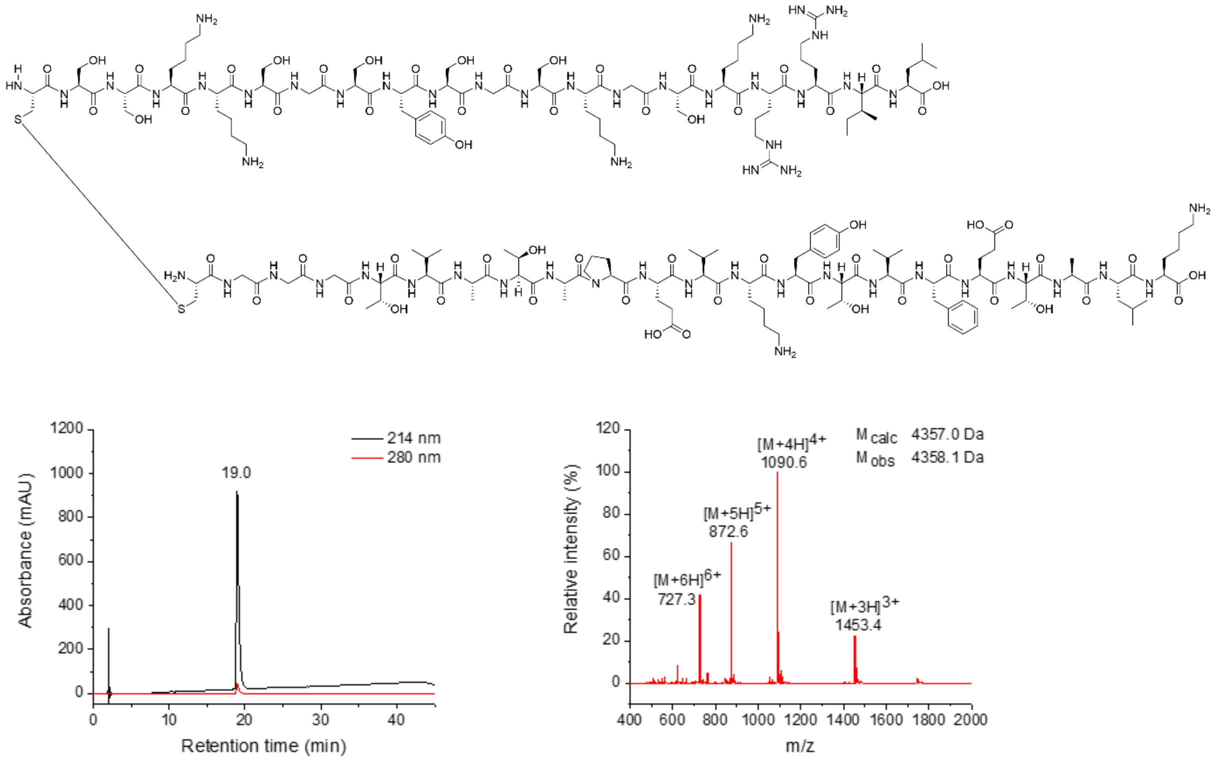

Figure S12 | Final analysis of the CysR5-Phl p 5 peptide-antigen conjugate. The analytical HPLC chromatogram (left) and the ESI-MS spectrum (right) of the purified conjugate.

The RRIL-1-P467 conjugate **2a** was synthesized successfully as described and obtained in 68% yield from theory. The analytical data is shown in Fig.S13. The mass spectrum shows the seven peaks for the $[M+10H]^{10+}$ (m/z (calc.): 694.60), $[M+9H]^{9+}$ (m/z (calc.): 771.67), $[M+8H]^{8+}$ (m/z (calc.): 868.00), $[M+7H]^{7+}$ (m/z (calc.): 991.86), $[M+6H]^{6+}$ (m/z (calc.): 1157.00), $[M+5H]^{5+}$ (m/z (calc.): 1388.20) and $[M+4H]^{4+}$ (m/z (calc.): 1735.00). The HPLC chromatogram shows a single peak with a retention time of 22.2 min indicting that the peptide was obtained in high (>95%) purity.

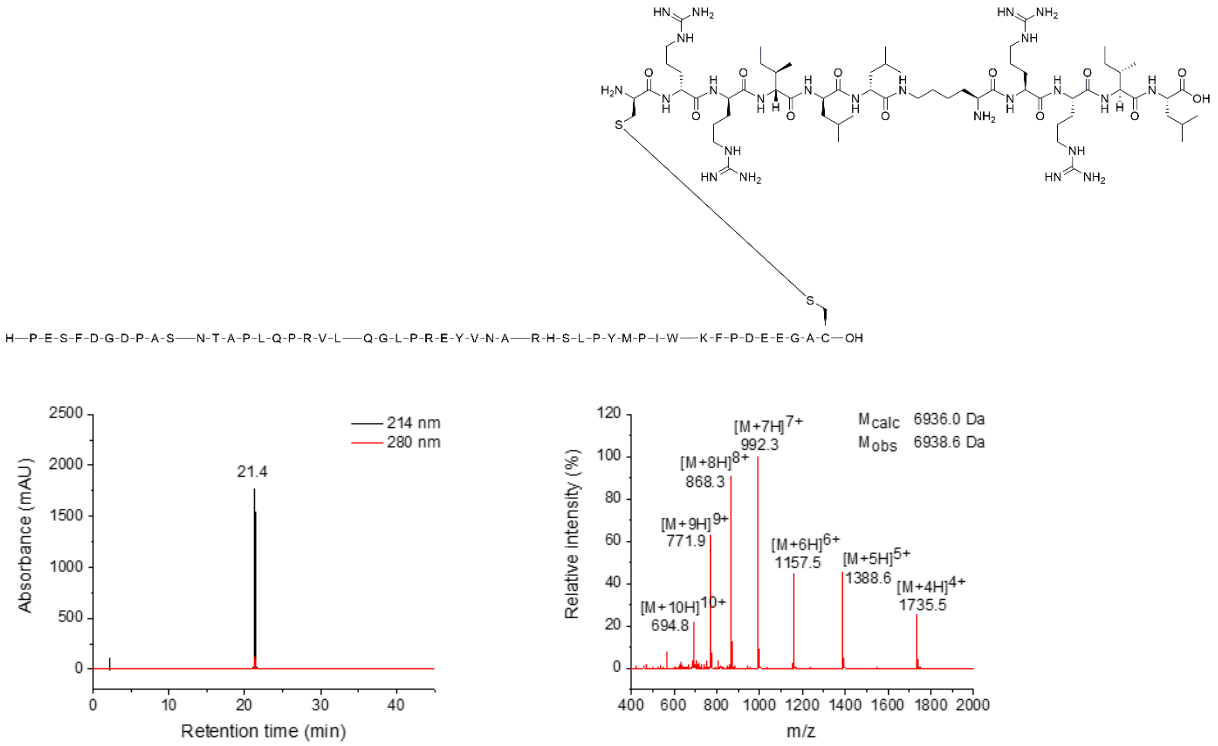

Figure S13 | Final analysis of the RRIL-1-P467 peptide-antigen conjugate. The analytical HPLC chromatogram (left) and the ESI-MS spectrum (right) of the purified conjugate.

The RRIL-1-Bet v 1 conjugate **2b** was synthesized successfully as described and obtained in 36% yield from theory. The analytical data is shown in Fig.S14. The mass spectrum shows the four peaks for the $[M+5H]^{5+}$ (m/z (calc.): 617.16), $[M+4H]^{4+}$ (m/z (calc.): 771.20), $[M+3H]^{3+}$ (m/z (calc.): 1027.93) and $[M+2H]^{2+}$ (m/z (calc.): 1541.40). The HPLC chromatogram shows a single peak with a retention time of 22.2 min indicting that the peptide was obtained in high (>95%) purity.

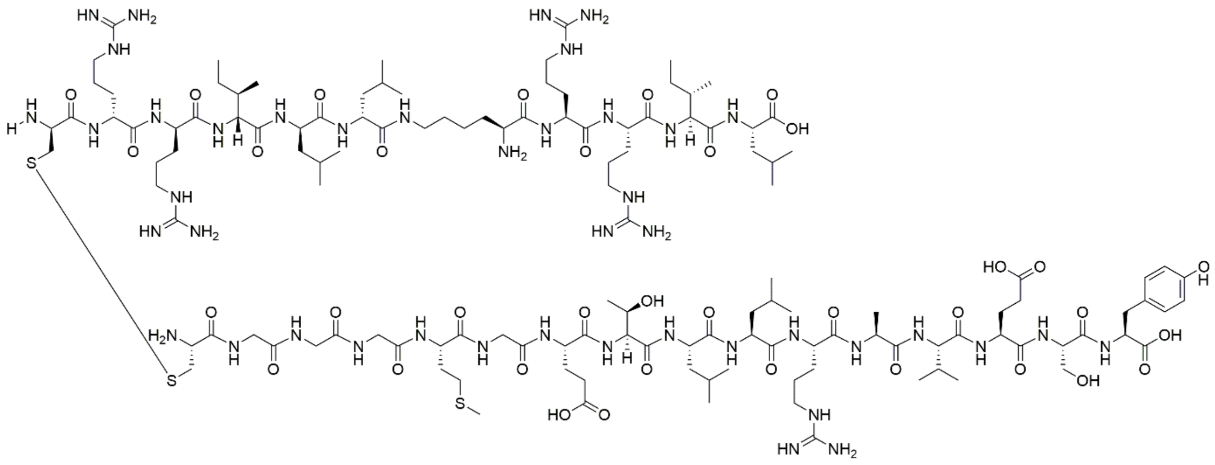

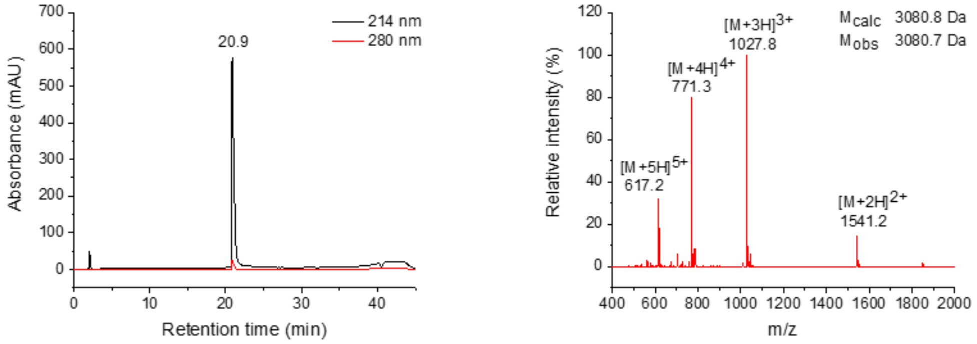

Figure S14 | Final analysis of the RRIL-1-Bet v 1peptide-antigen conjugate. The analytical HPLC chromatogram (left) and the ESI-MS spectrum (right) of the purified conjugate.

The RRIL-1-Phl p 5 conjugate **2c** was synthesized successfully as described and obtained in 29% yield from theory. The analytical data is shown in Fig.S15. The mass spectrum shows the five peaks for the $[M+6H]^{6+}$ (m/z (calc.): 614.42), $[M+5H]^{5+}$ (m/z (calc.): 737.10), $[M+4H]^{4+}$ (m/z (calc.): 921.13), $[M+3H]^{3+}$ (m/z (calc.): 1227.83) and $[M+2H]^{2+}$ (m/z (calc.): 1841.25). The HPLC chromatogram shows a single peak with a retention time of 22.2 min indicting that the peptide was obtained in high (>95%) purity.

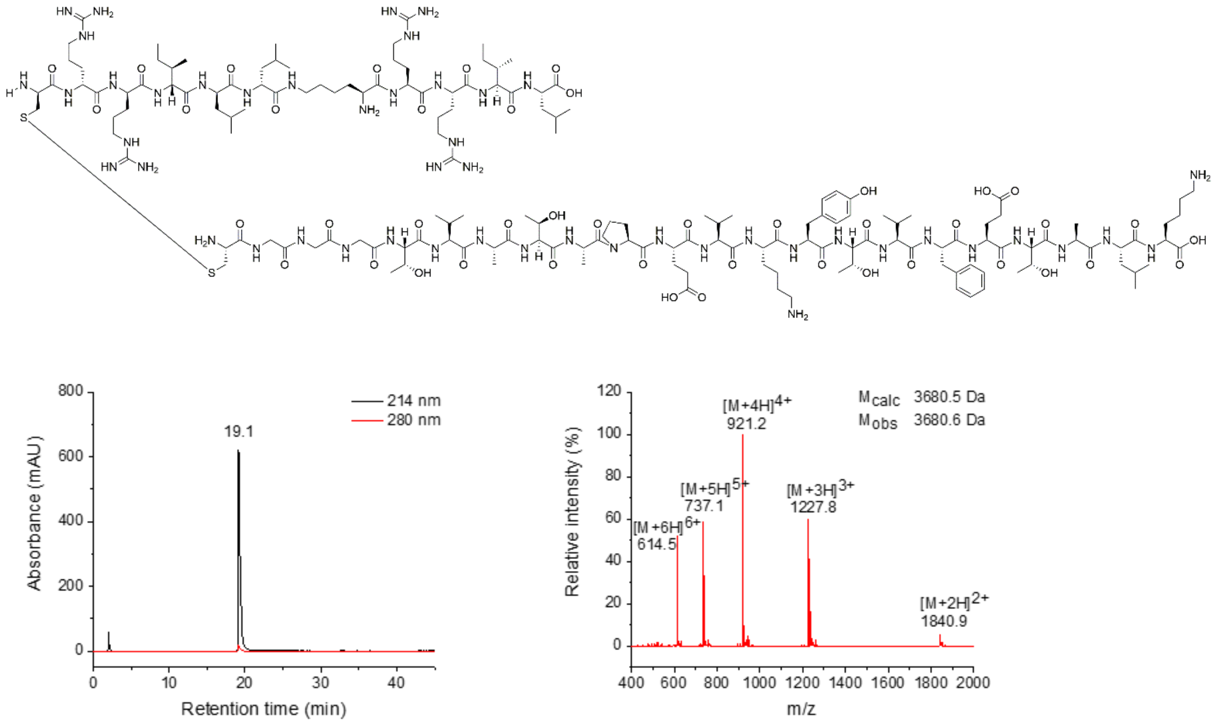

FigureS15 | Final analysis of the RRIL-1-Phl p 5 peptide-antigen conjugate. The analytical HPLC chromatogram (left) and the ESI-MS spectrum (right) of the purified conjugate.

The RRIL-2-P467 conjugate **3a** was synthesized successfully as described and obtained in 82% yield from theory. The analytical data is shown in Fig.S16. The mass spectrum shows the seven peaks for the $[M+10H]^{10+}$ (m/z (calc.): 694.60), $[M+9H]^{9+}$ (m/z (calc.): 771.67), $[M+8H]^{8+}$ (m/z (calc.): 868.00), $[M+7H]^{7+}$ (m/z (calc.): 991.86), $[M+6H]^{6+}$ (m/z (calc.): 1157.00), $[M+5H]^{5+}$ (m/z (calc.): 1388.20) and $[M+4H]^{4+}$ (m/z (calc.): 1735.00). The HPLC chromatogram shows a single peak with a retention time of 22.2 min indicting that the peptide was obtained in high (>95%) purity.

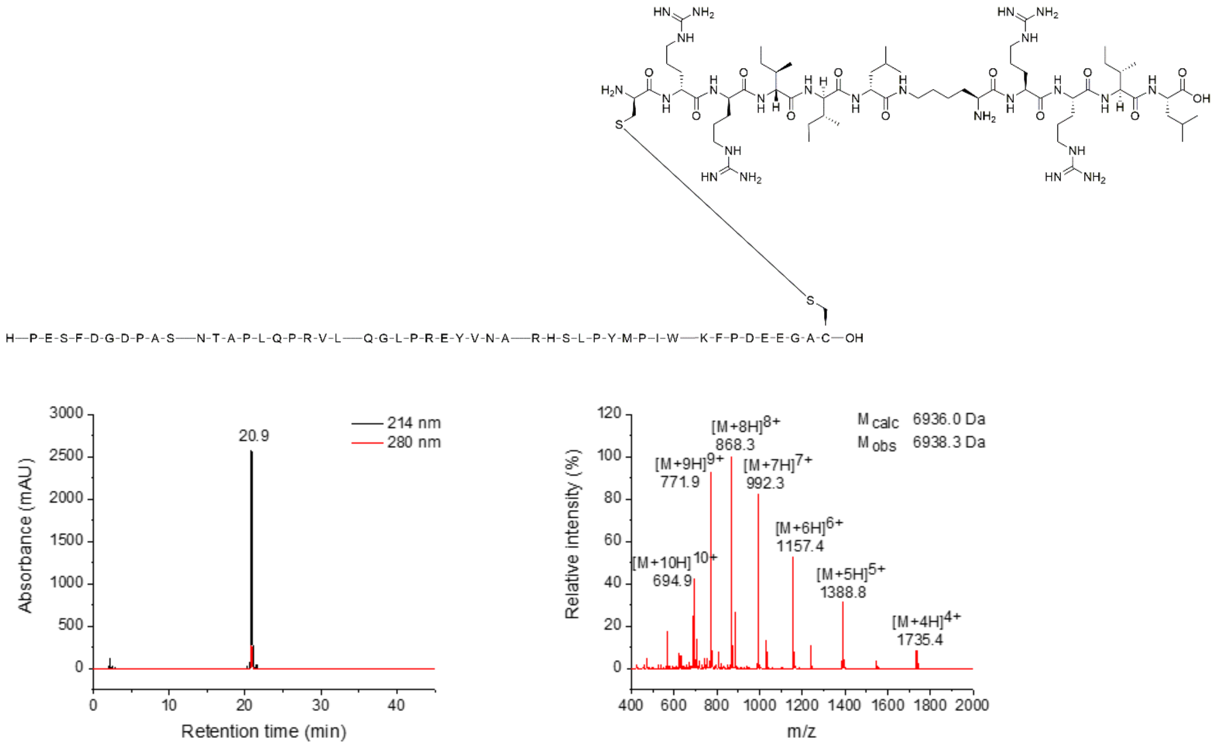

Figure S16 | Final analysis of the RRIL-2-P467 peptide-antigen conjugate. The analytical HPLC chromatogram (left) and the ESI-MS spectrum (right) of the purified conjugate.

The RRIL-2-Bet v 1 conjugate **3b** was synthesized successfully as described and obtained in 60% yield from theory. The analytical data is shown in Fig.S17. The mass spectrum shows the four peaks for the $[M+5H]^{5+}$ (m/z (calc.): 617.16), $[M+4H]^{4+}$ (m/z (calc.): 771.20), $[M+3H]^{3+}$ (m/z (calc.): 1027.93) and $[M+2H]^{2+}$ (m/z (calc.): 1541.40). The HPLC chromatogram shows a single peak with a retention time of 22.2 min indicting that the peptide was obtained in high (>95%) purity.

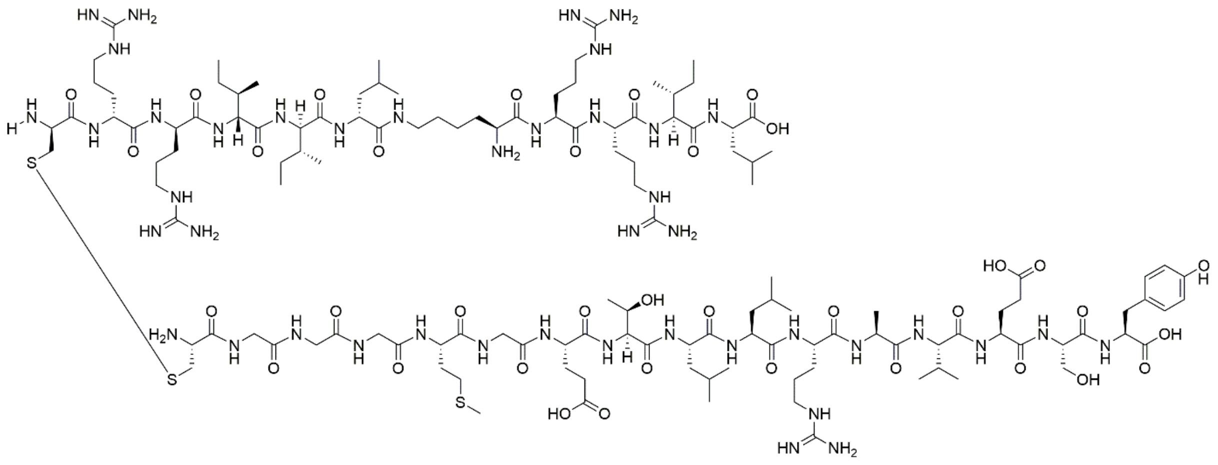

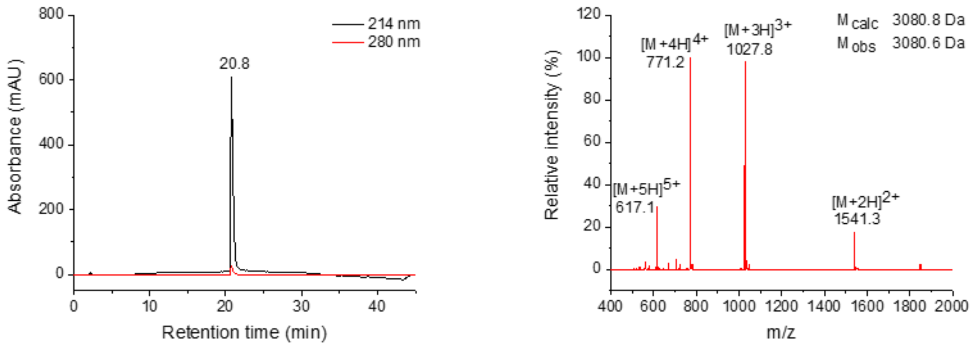

Figure S17 | Final analysis of the RRIL-2-Bet v 1peptide-antigen conjugate. The analytical HPLC chromatogram (left) and the ESI-MS spectrum (right) of the purified conjugate.

The RRIL-2-Phl p 5 conjugate **3c** was synthesized successfully as described and obtained in 33% yield from theory. The analytical data is shown in Fig.S18. The mass spectrum shows the five peaks for the $[M+6H]6^{+}$ (m/z (calc.): 614.42), $[M+5H]^{5+}$ (m/z (calc.): 737.10), $[M+4H]^{4+}$ (m/z (calc.): 921.13), $[M+3H]^{3+}$ (m/z (calc.): 1227.83) and $[M+2H]^{2+}$ (m/z (calc.): 1841.25). The HPLC chromatogram shows a single peak with a retention time of 22.2 min indicting that the peptide was obtained in high (>95%) purity.

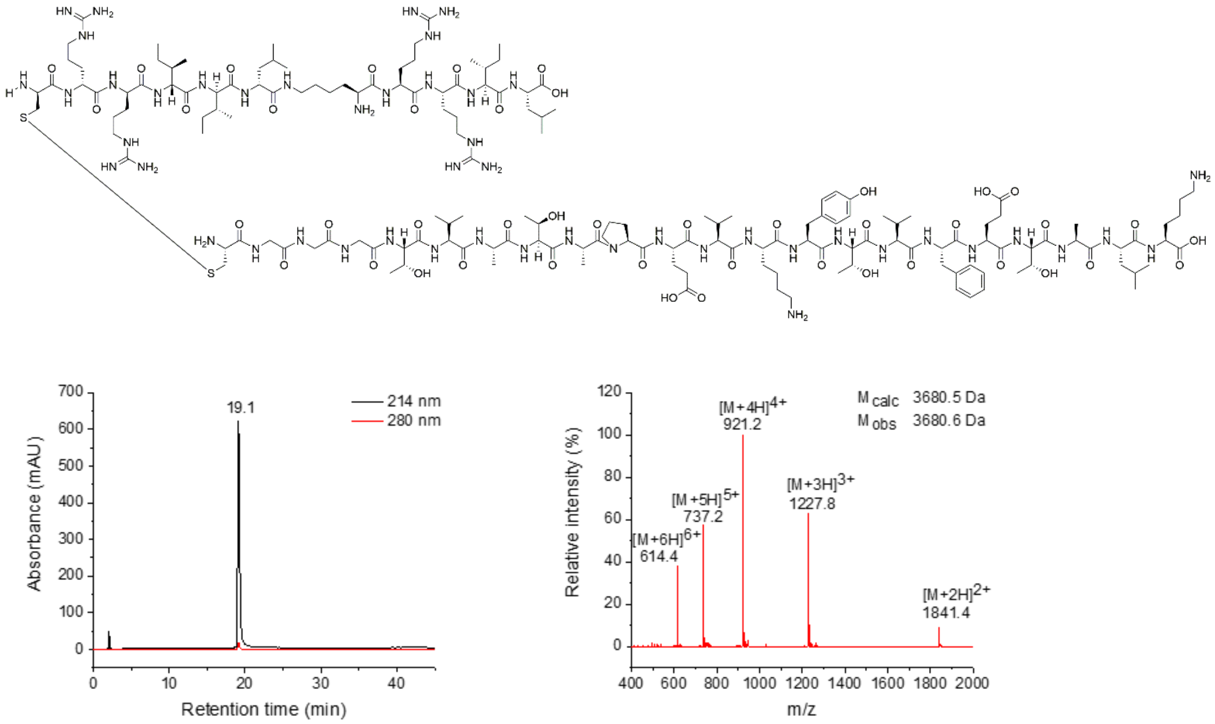

Figure S18 | Final analysis of the RRIL-2-Phl p 5 peptide-antigen conjugate. The analytical HPLC chromatogram (left) and the ESI-MS spectrum (right) of the purified conjugate.

The RRLL-P467 conjugate **4a** was synthesized successfully as described and obtained in 68% yield from theory. The analytical data is shown in Fig.S19. The mass spectrum shows the seven peaks for the $[M+10H]^{10+}$ (m/z (calc.): 694.60), $[M+9H]^{9+}$ (m/z (calc.): 771.67), $[M+8H]^{8+}$ (m/z (calc.): 868.00), $[M+7H]^{7+}$ (m/z (calc.): 991.86), $[M+6H]^{6+}$ (m/z (calc.): 1157.00), $[M+5H]^{5+}$ (m/z (calc.): 1388.20) and $[M+4H]^{4+}$ (m/z (calc.): 1735.00). The HPLC chromatogram shows a single peak with a retention time of 22.2 min indicting that the peptide was obtained in high (>95%) purity.

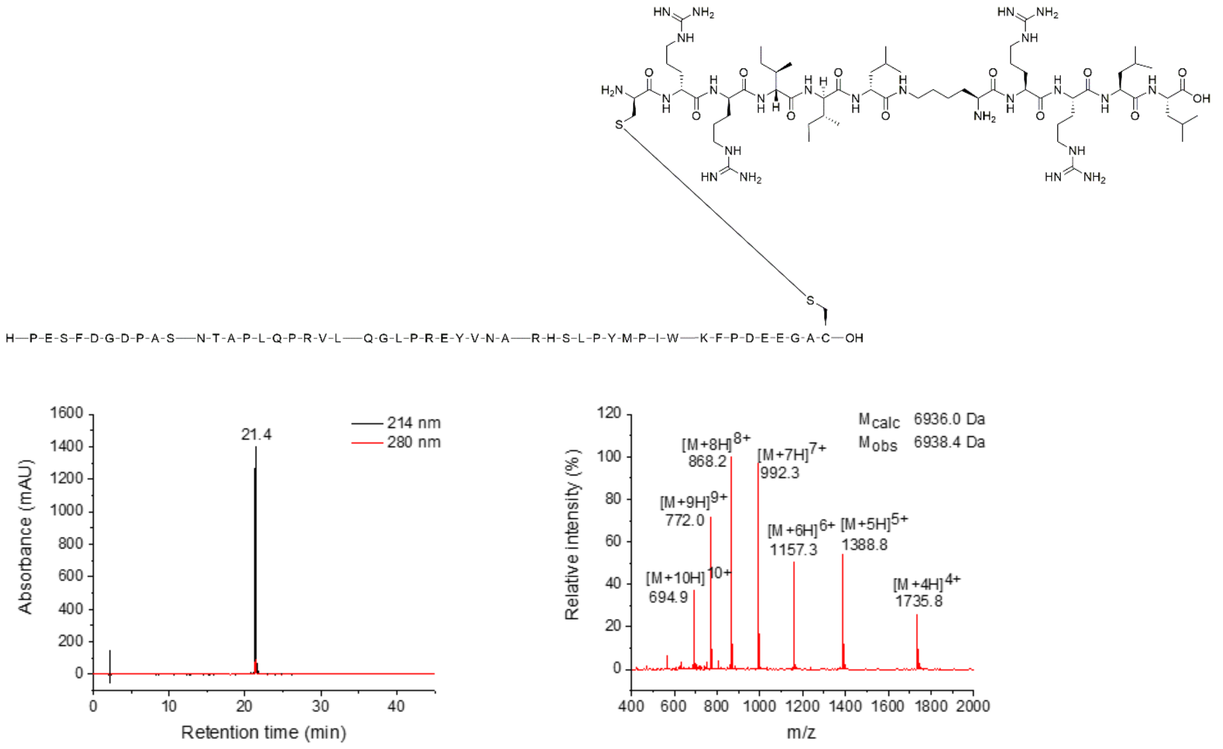

Figure S19 | Final analysis of the RRLL-P467 peptide-antigen conjugate. The analytical HPLC chromatogram (left) and the ESI-MS spectrum (right) of the purified conjugate.

The RRLL-Bet v 1 conjugate **4b** was synthesized successfully as described and obtained in 39% yield from theory. The analytical data is shown in Fig.S20. The mass spectrum shows the four peaks for the $[M+5H]^{5+}$ (m/z (calc.): 617.16), $[M+4H]^{4+}$ (m/z (calc.): 771.20), $[M+3H]^{3+}$ (m/z (calc.): 1027.93) and $[M+2H]^{2+}$ (m/z (calc.): 1541.40). The HPLC chromatogram shows a single peak with a retention time of 22.2 min indicting that the peptide was obtained in high (>95%) purity.

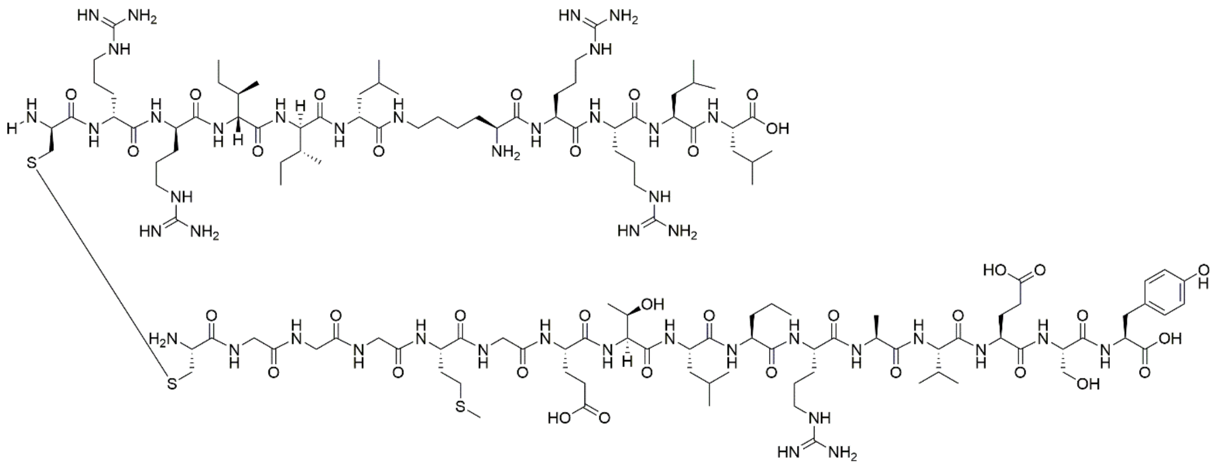

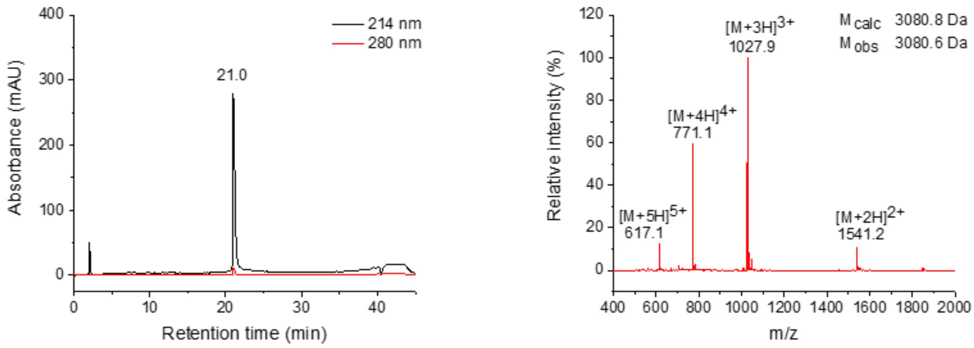

Figure S20 | Final analysis of the RRLL-Bet v 1peptide-antigen conjugate. The analytical HPLC chromatogram (left) and the ESI-MS spectrum (right) of the purified conjugate.

The RRLL-Phl p 5 conjugate **4c** was synthesized successfully as described and obtained in 39% yield from theory. The analytical data is shown in Fig.S21. The mass spectrum shows the five peaks for the $[M+6H]6^{+}$ (m/z (calc.): 614.42), $[M+5H]^{5+}$ (m/z (calc.): 737.10), $[M+4H]^{4+}$ (m/z (calc.): 921.13), $[M+3H]^{3+}$ (m/z (calc.): 1227.83) and $[M+2H]^{2+}$ (m/z (calc.): 1841.25). The HPLC chromatogram shows a single peak with a retention time of 19.2 min indicting that the peptide was obtained in high (>95%) purity.

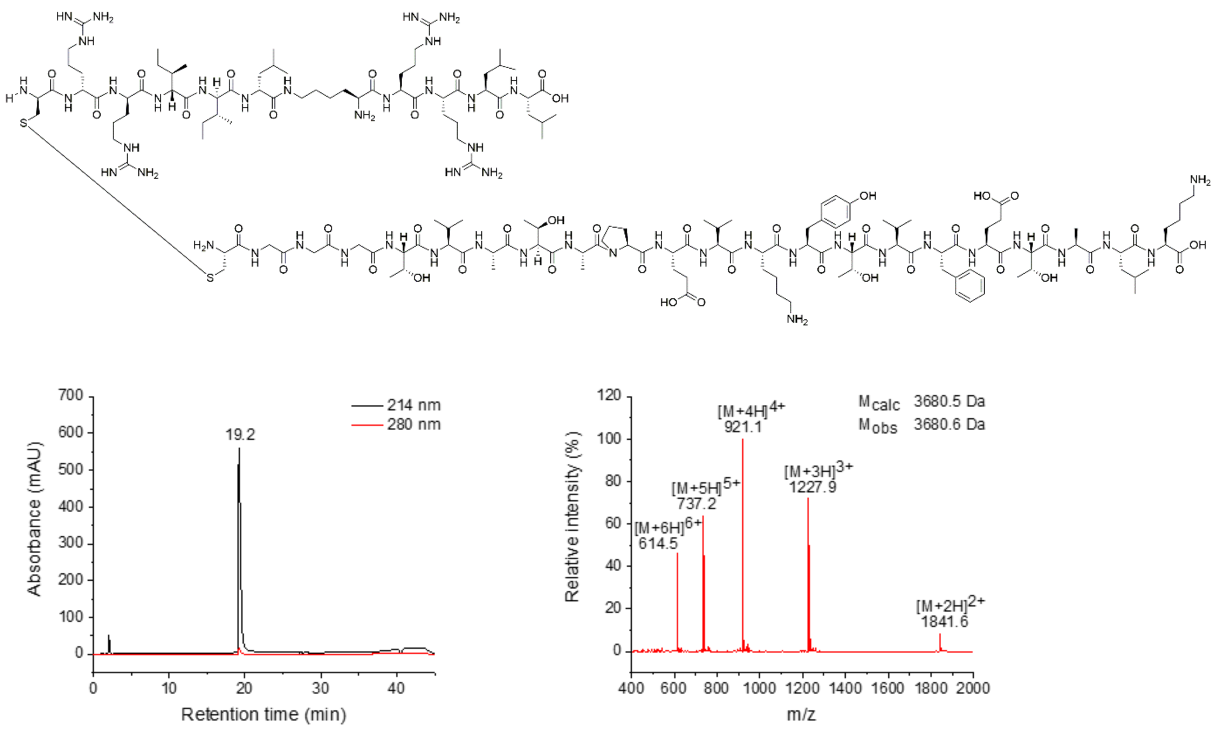

Figure S21 | Final analysis of the RRLL-Phl p 5 peptide-antigen conjugate. The analytical HPLC chromatogram (left) and the ESI-MS spectrum (right) of the purified conjugate.

Release of peptide conjugate from silica particles

Particles were synthesized from 1.895 mL of a CysR5-Phl p 5 **1c** solution as described above. The particles were distributed in equal amounts into Eppendorf tubes and 50 µl of either 50 mM potassium phosphate buffer pH 4 or pH 7 was added. The particles were suspended by pipetting up and down and vortexing. Then, they were incubated at 37 °C on the shaker (600 rpm). After 1, 3, 10, and 11 h, the sample was centrifuged (14000 rpm, 5 min, room temperature) and 45 µl of the supernatant were transferred into a HPLC vial that already contained 45 µl guanidinium chloride buffer (6 M GuHCl, 50 mM Tris-HCl pH 7.5). The resulting 90 µl were injected into the HPLC system. The time points were measured in triplicates. By comparing the area under the curve (AUC) of the peptide conjugate at the different time points with the AUC of a peptide conjugate standard with a known concentration, the relative release was determined.

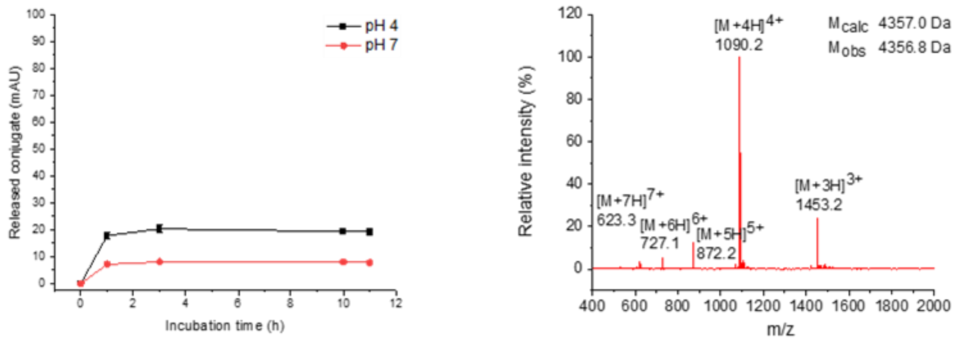

Figure S22. Release of CysR5-Phl p 5 peptide-antigen conjugate **1c** from silica particles. Fraction released (left) at pH 4 and pH 7. ESI-MS spectrum (right) of the conjugate after 11 h incubation at pH 4.

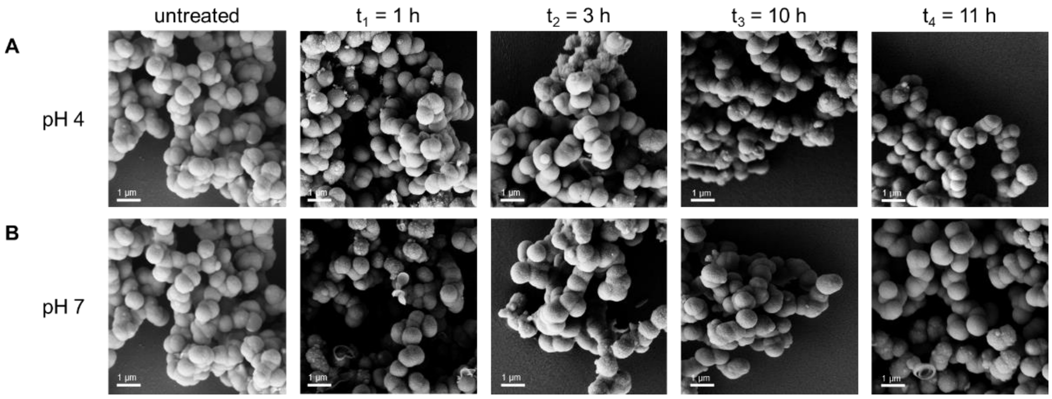

Figure S23. Scanning electron micrographs (SEM) of silica particles before and after incubation at multiple time points (t_x) in phosphate buffer at pH 4(A) and pH 7 (B).

Zeta potential

Stable aqueous suspensions of the material (0.3 mg/ml) were obtained by performing three cycles of vortex shaking (10 minutes) and ultrasonic bath treatment (20 minutes). The sample suspension was then analyzed from pH 4 to pH 8 using a Malvern Zetasizer Nano ZS. The vertical line represents the isoelectric point (pI).

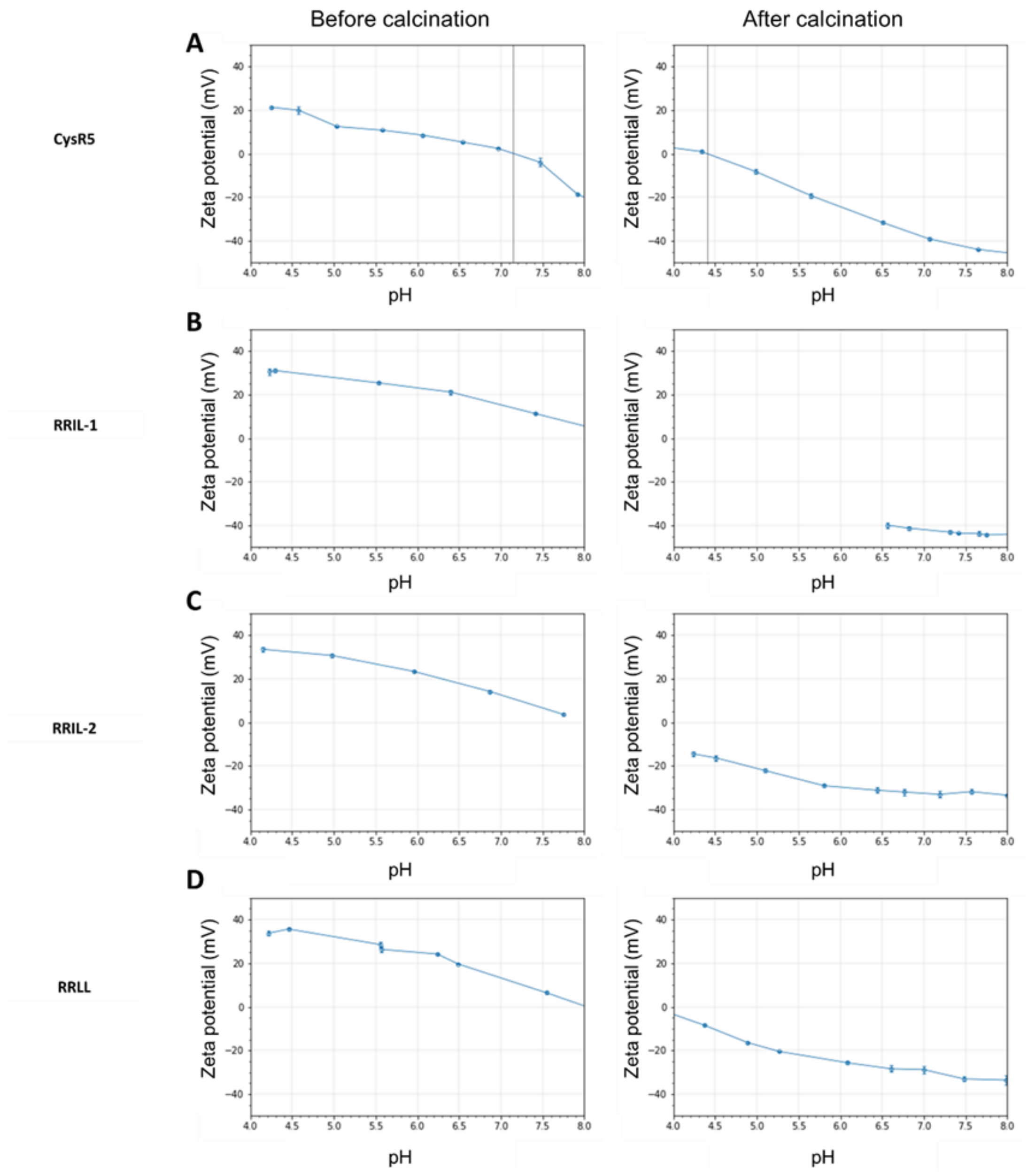

Figure S24. Zeta potential of CysR5 (**1'**), RRIL-1 (**2'**), RRIL-2 (**3'**) and RRLL (**4'**) silica particles, before and after calcination, over a range from pH 4 to pH 8.

Pore size distribution (NLDFT method) derived from nitrogen physisorption analysis

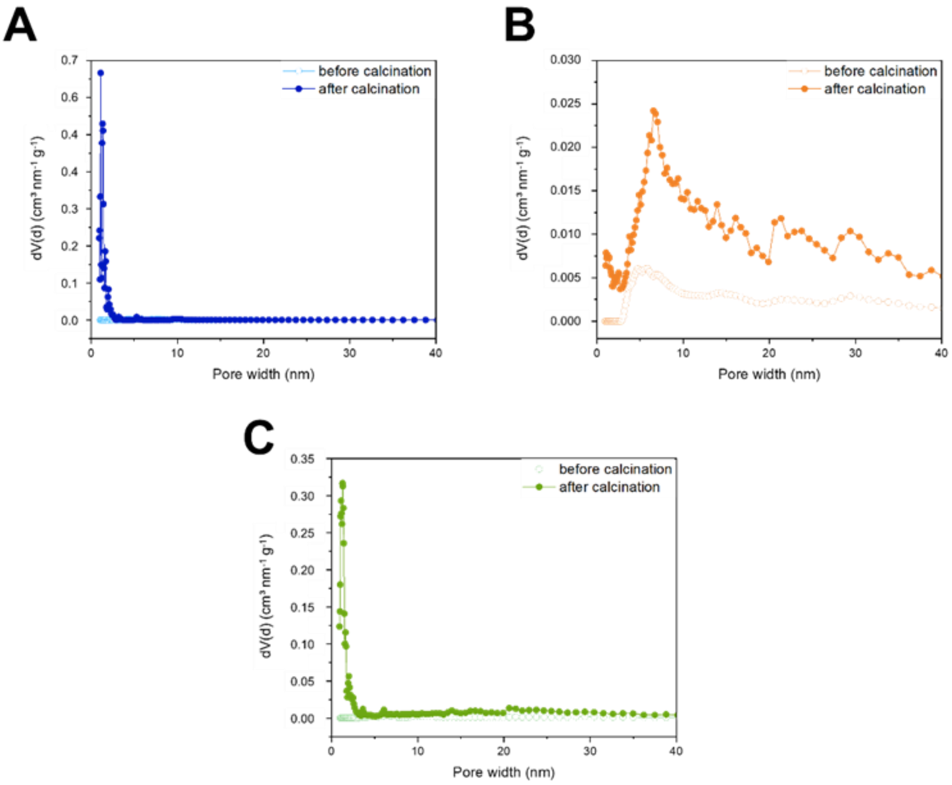

Figure S25. Pore size distribution (NLDFT method) derived from nitrogen physisorption analysis for CysR5 particles in blue (A), RRIL-1 particles in orange (B) and RRIL-2 particles in green (C) at −196°C before (hollow circle) and after (filled circle) calcination.

Peptide characteristics

Table S1. Calculated via ProtParam by Expasy

Peptide	Number of amino acids	Molecular weight / g·mol^{-1}	Theo. pI	Negative charges	Positive charges	Total charge	Hydrophobic residues / %
LL-37	37	4493.32	10.61	5	11	6	35.1
CysR5	20	2116.42	10.58	0	6	6	15.0
RRIL-1	11	1439.88	12.00	0	5	5	45.5
